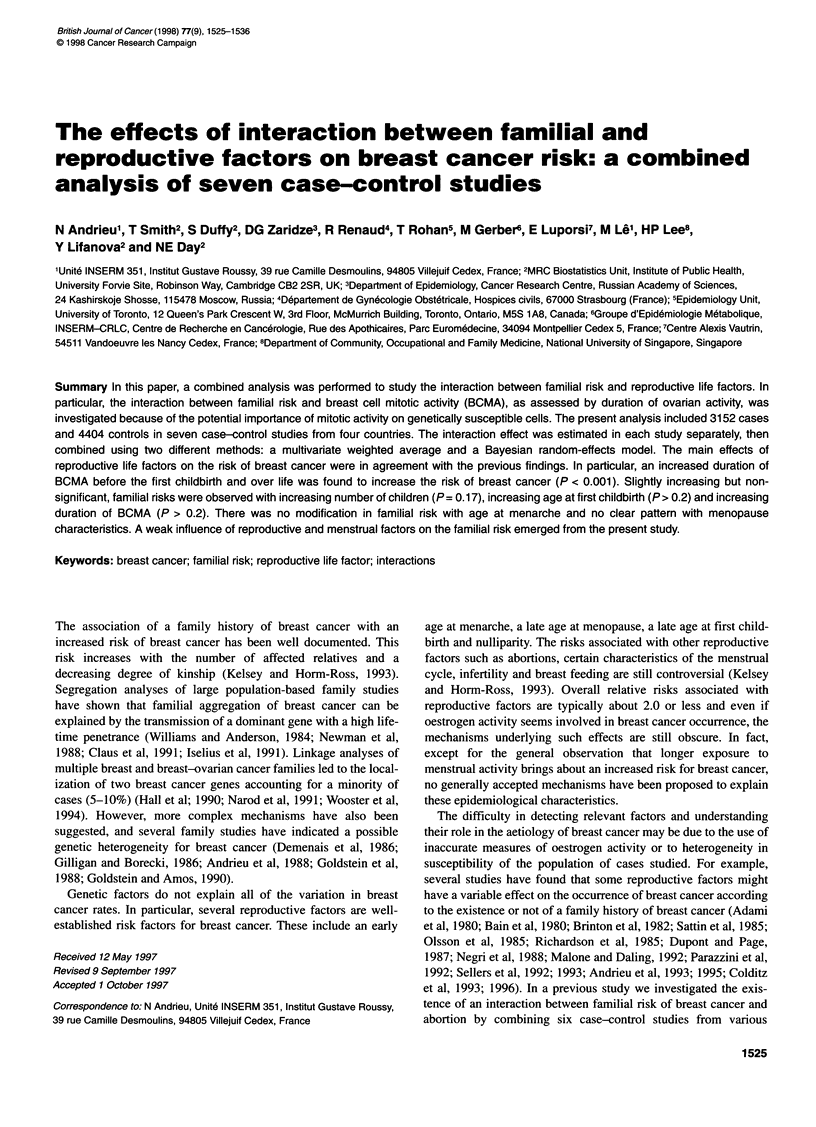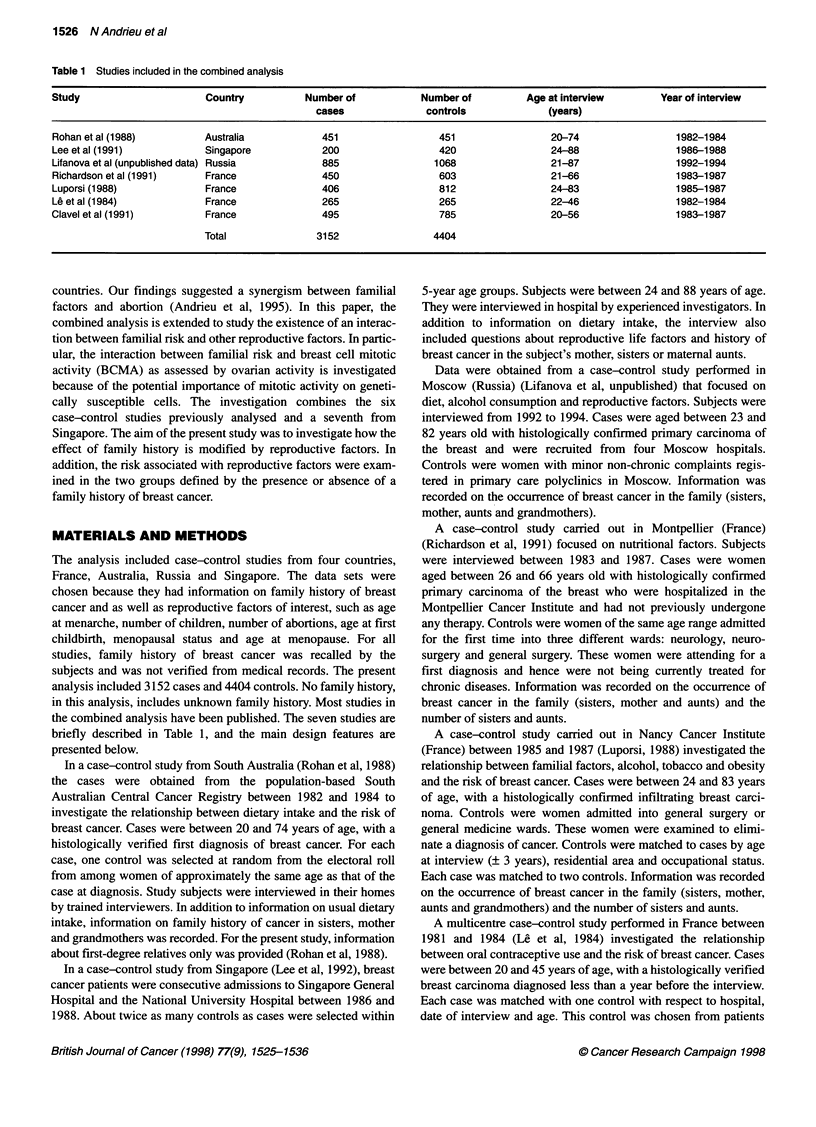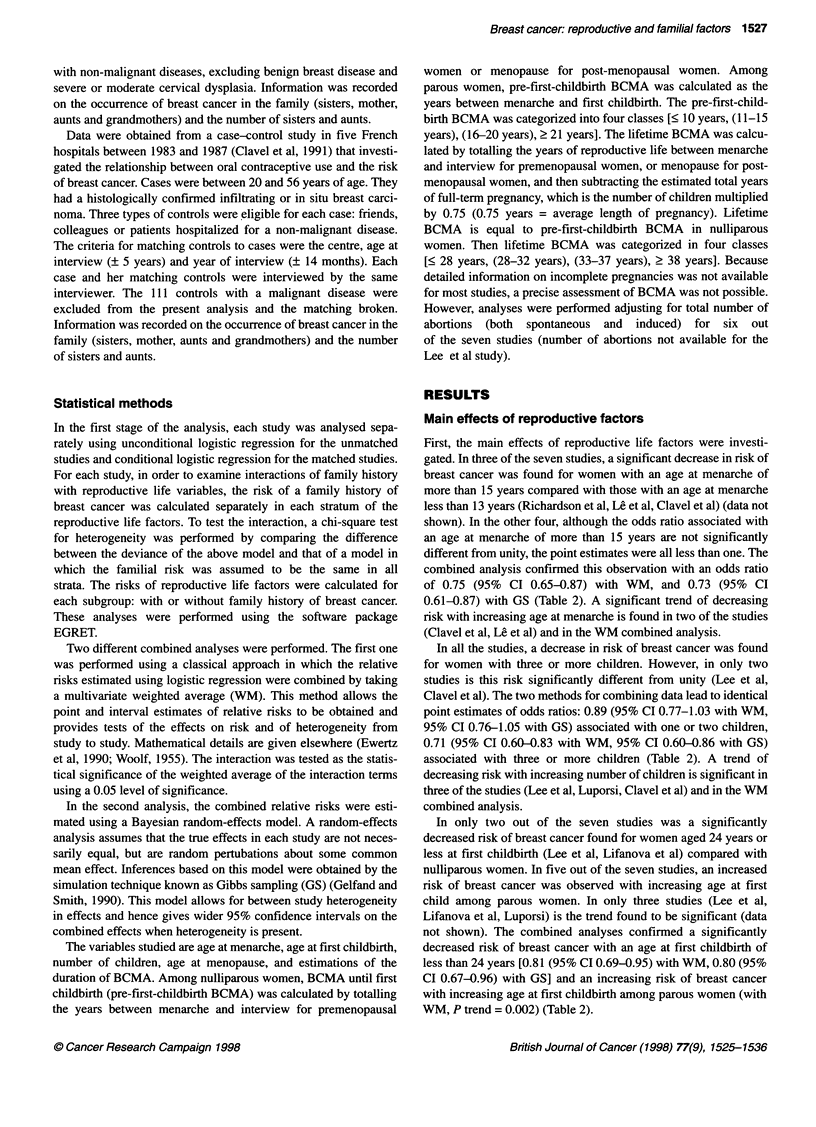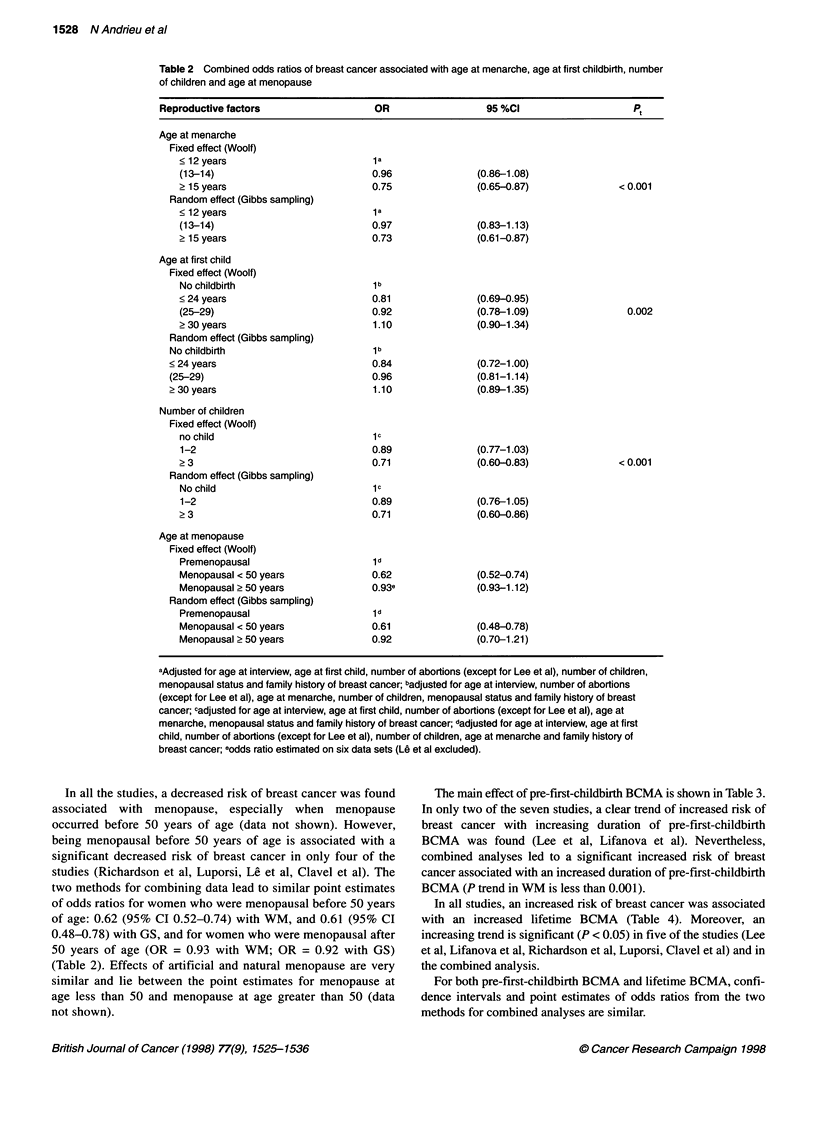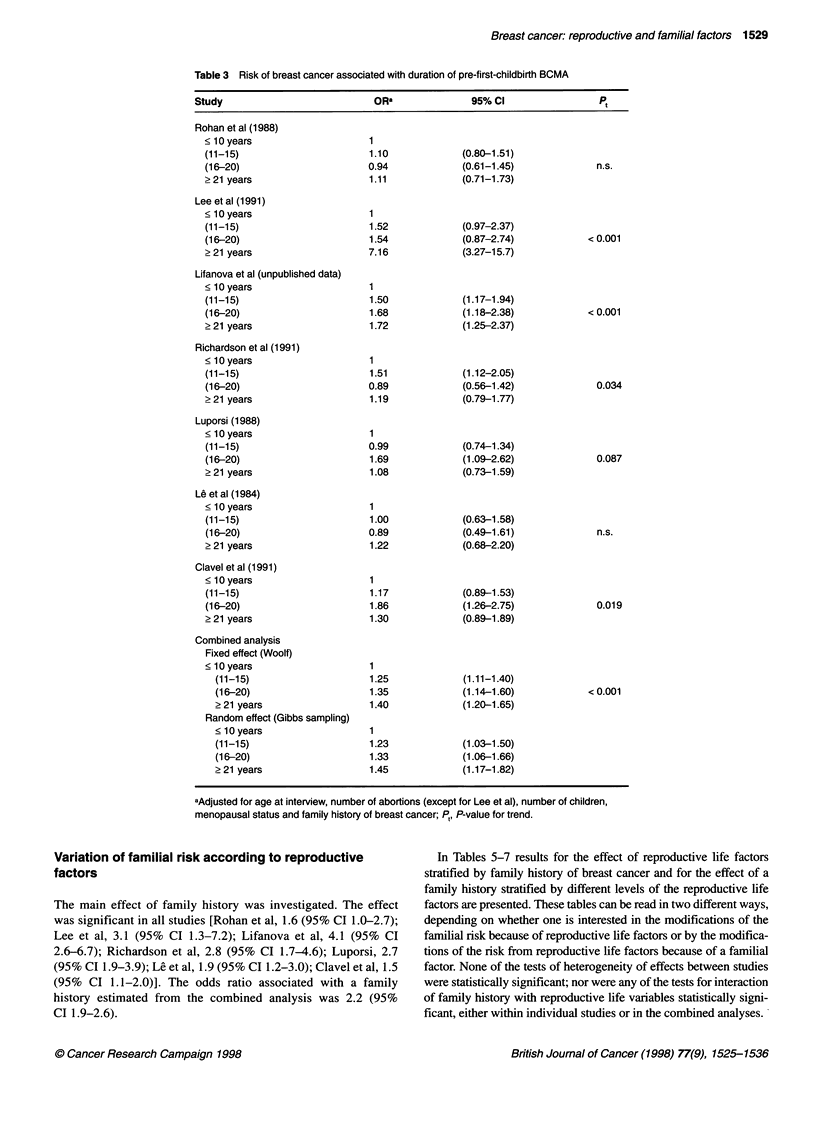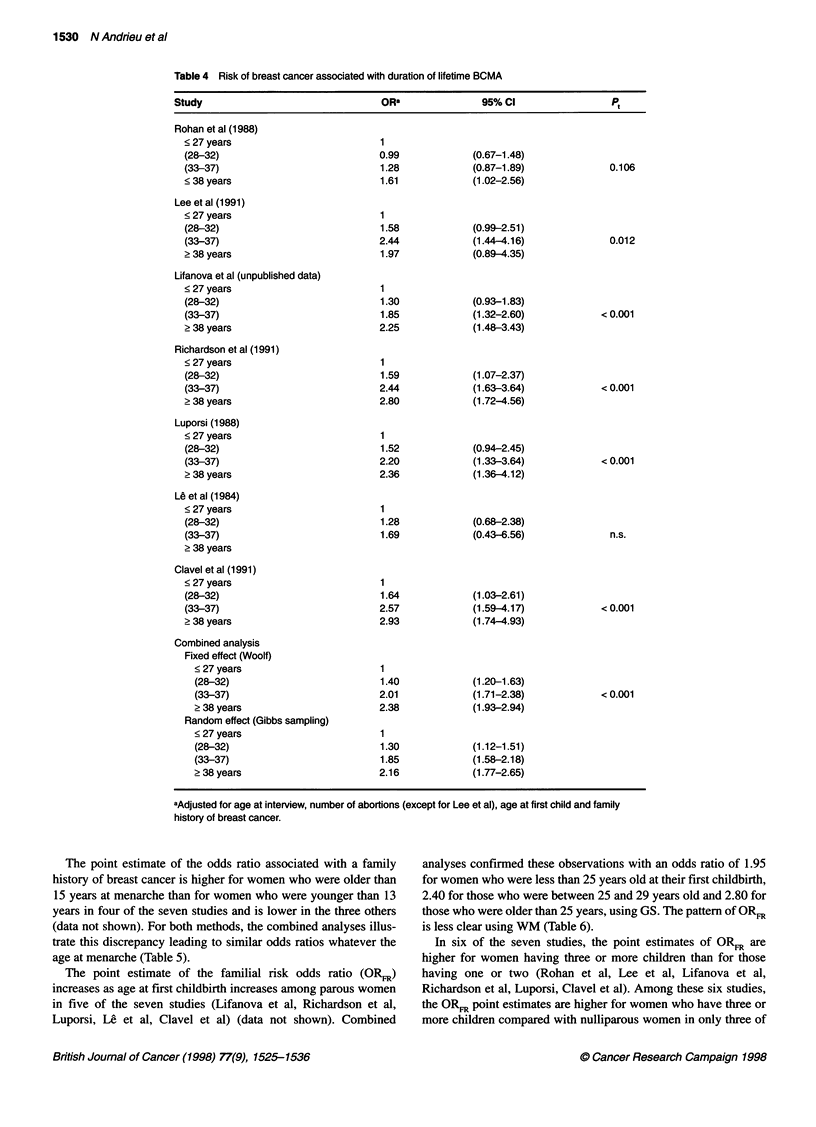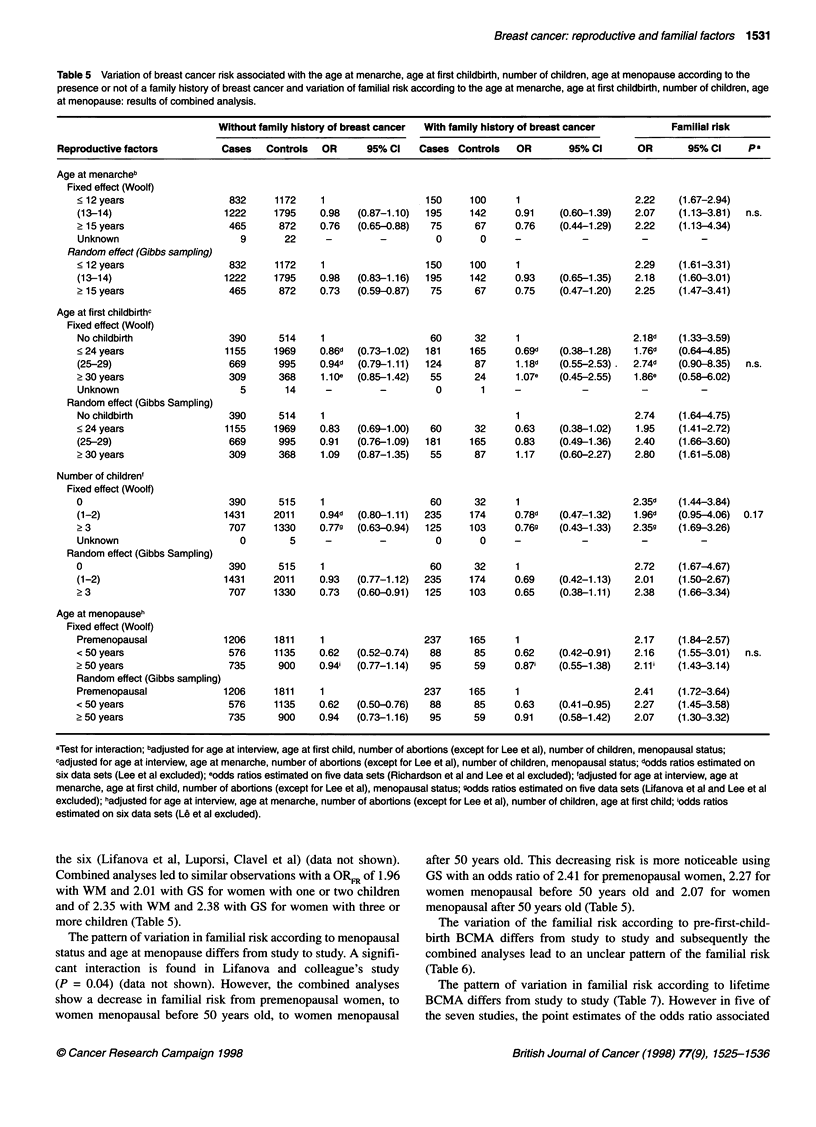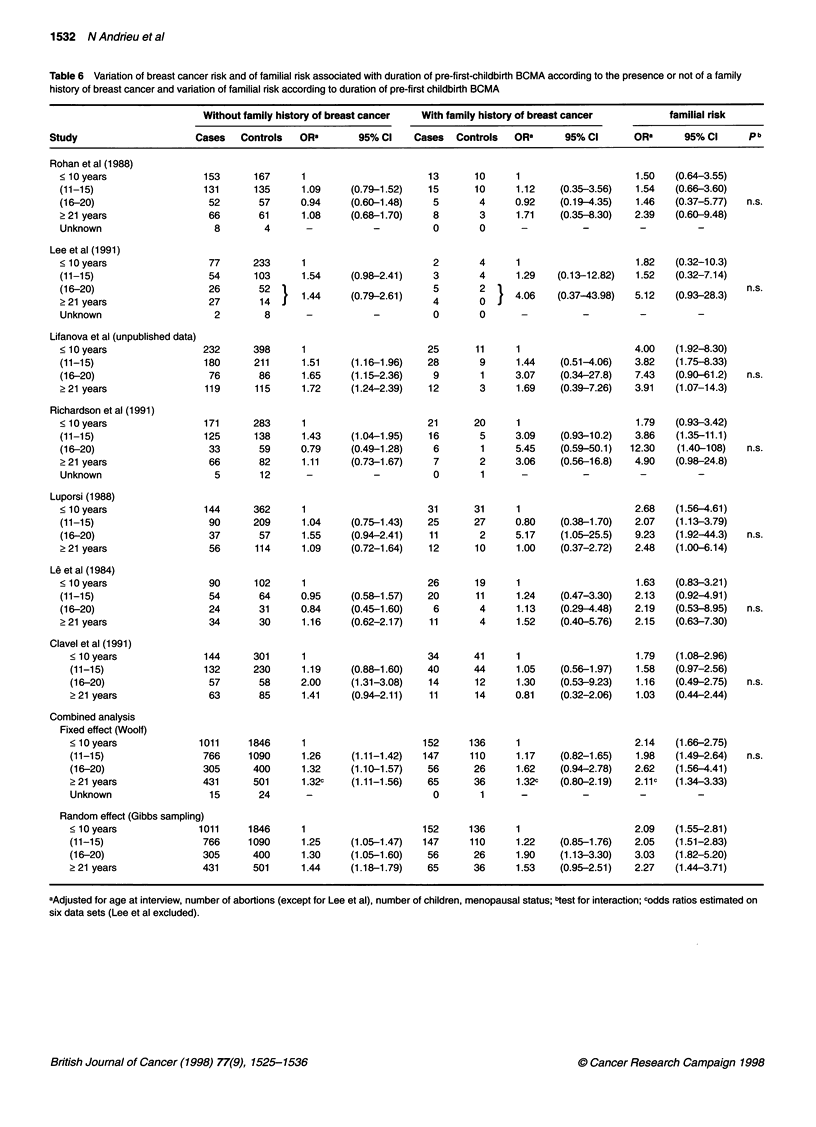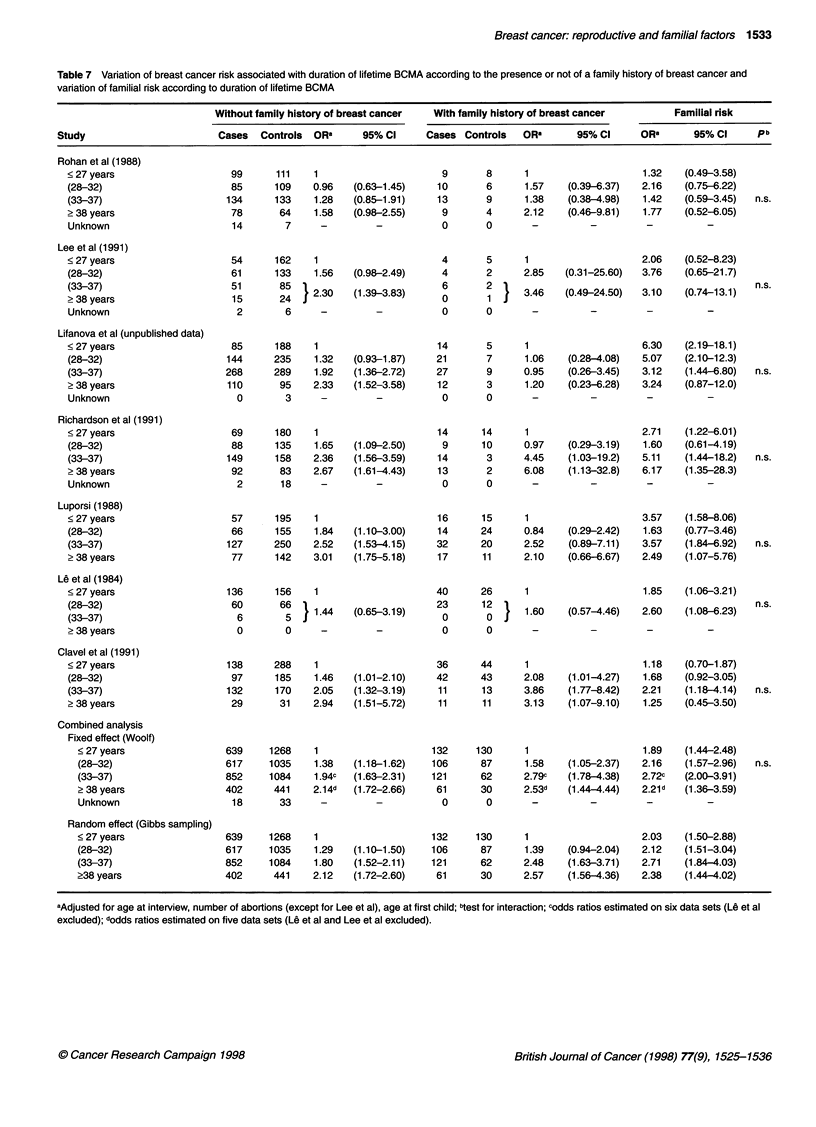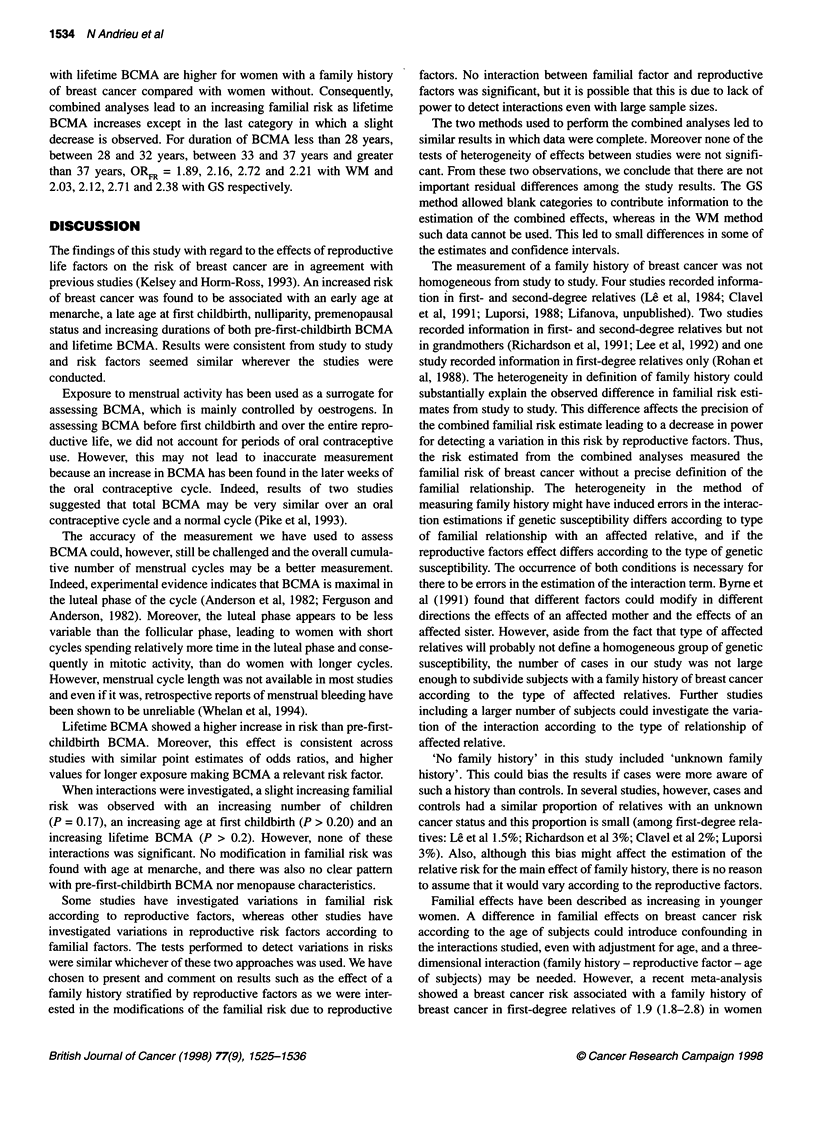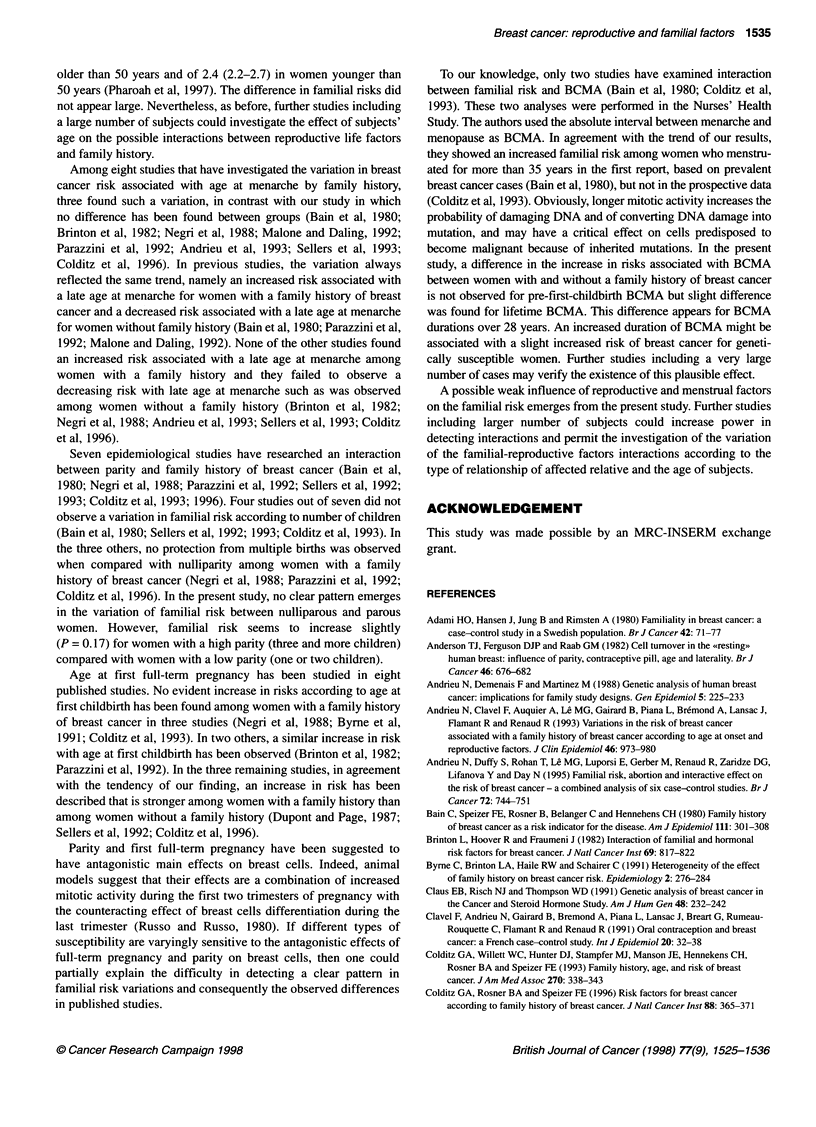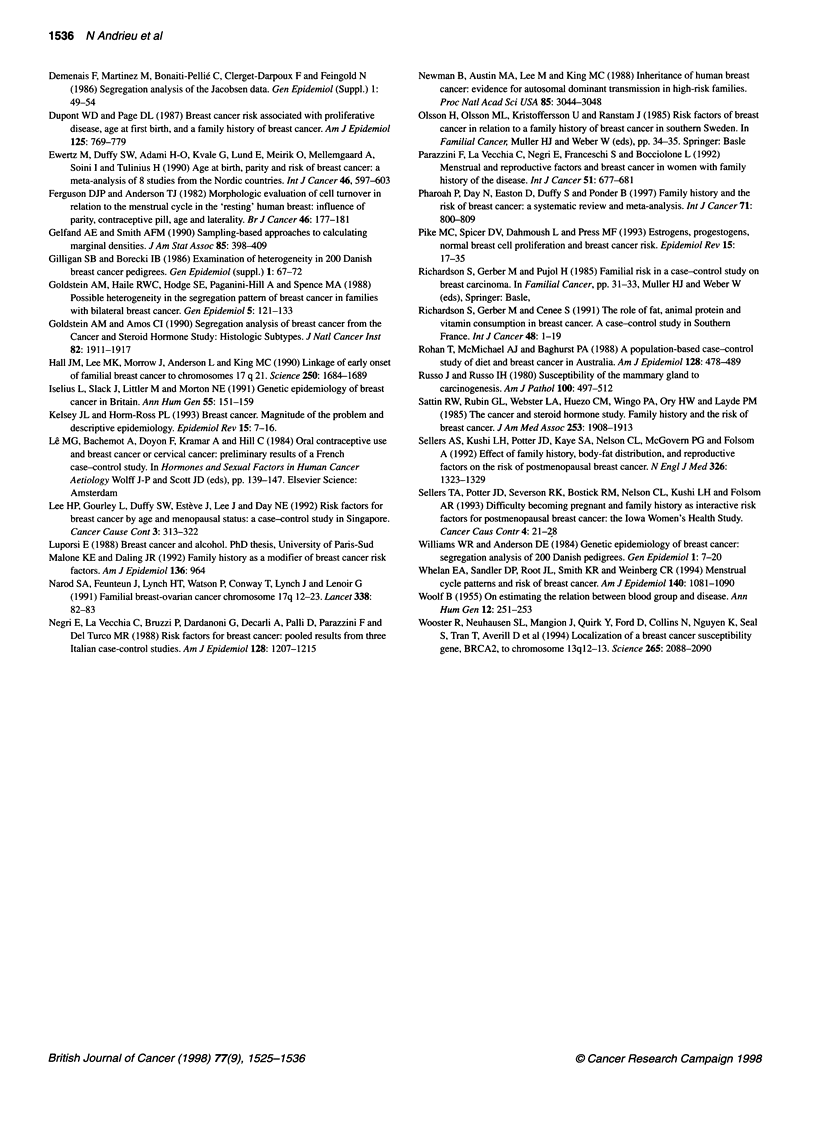# The effects of interaction between familial and reproductive factors on breast cancer risk: a combined analysis of seven case-control studies.

**DOI:** 10.1038/bjc.1998.251

**Published:** 1998-05

**Authors:** N. Andrieu, T. Smith, S. Duffy, D. G. Zaridze, R. Renaud, T. Rohan, M. Gerber, E. Luporsi, M. LÃª, H. P. Lee, Y. Lifanova, N. E. Day

**Affiliations:** UnitÃ© INSERM 351, Institut Gustave Roussy, Villejuif, France.

## Abstract

In this paper, a combined analysis was performed to study the interaction between familial risk and reproductive life factors. In particular, the interaction between familial risk and breast cell mitotic activity (BCMA), as assessed by duration of ovarian activity, was investigated because of the potential importance of mitotic activity on genetically susceptible cells. The present analysis included 3152 cases and 4404 controls in seven case-control studies from four countries. The interaction effect was estimated in each study separately, then combined using two different methods: a multivariate weighted average and a Bayesian random-effects model. The main effects of reproductive life factors on the risk of breast cancer were in agreement with the previous findings. In particular, an increased duration of BCMA before the first childbirth and over life was found to increase the risk of breast cancer (P < 0.001). Slightly increasing but non-significant, familial risks were observed with increasing number of children (P = 0.17), increasing age at first childbirth (P > 0.2) and increasing duration of BCMA (P > 0.2). There was no modification in familial risk with age at menarche and no clear pattern with menopause characteristics. A weak influence of reproductive and menstrual factors on the familial risk emerged from the present study.


British Journal of Cancer (1998) 77(9), 1525–1536
© 1998 Cancer Research Campaign

### The effects of interaction between familial and reproductive factors on breast cancer risk: a combined analysis of seven case–control studies

**N Andrieu[1], T Smith[2], S Duffy[2], DG Zaridze[3], R Renaud[4], T Rohan[5], M Gerber[6], E Luporsi[7], M Lê[1], HP Lee[8], Y Lifanova[2] and NE Day[2]**

[1]Unité INSERM 351, Institut Gustave Roussy, 39 rue Camille Desmoulins, 94805 Villejuif Cedex, France; [2]MRC Biostatistics Unit, Institute of Public Health, University Forvie Site, Robinson Way, Cambridge CB2 2SR, UK; [3]Department of Epidemiology, Cancer Research Centre, Russian Academy of Sciences, 24 Kashirskoje Shosse, 115478 Moscow, Russia; [4]Département de Gynécologie Obstétricale, Hospices civils, 67000 Strasbourg (France); [5]Epidemiology Unit, University of Toronto, 12 Queen's Park Crescent W, 3rd Floor, McMurrich Building, Toronto, Ontario, M5S 1A8, Canada; [6]Groupe d'Epidémiologie Métabolique, INSERM–CRLC, Centre de Recherche en Cancérologie, Rue des Apothicaires, Parc Euromédecine, 34094 Montpellier Cedex 5, France; [7]Centre Alexis Vautrin, 54511 Vandoeuvre les Nancy Cedex, France; [8]Department of Community, Occupational and Family Medicine, National University of Singapore, Singapore

**Summary** In this paper, a combined analysis was performed to study the interaction between familial risk and reproductive life factors. In particular, the interaction between familial risk and breast cell mitotic activity (BCMA), as assessed by duration of ovarian activity, was investigated because of the potential importance of mitotic activity on genetically susceptible cells. The present analysis included 3152 cases and 4404 controls in seven case–control studies from four countries. The interaction effect was estimated in each study separately, then combined using two different methods: a multivariate weighted average and a Bayesian random-effects model. The main effects of reproductive life factors on the risk of breast cancer were in agreement with the previous findings. In particular, an increased duration of BCMA before the first childbirth and over life was found to increase the risk of breast cancer ($P < 0.001$). Slightly increasing but non-significant, familial risks were observed with increasing number of children ($P = 0.17$), increasing age at first childbirth ($P > 0.2$) and increasing duration of BCMA ($P > 0.2$). There was no modification in familial risk with age at menarche and no clear pattern with menopause characteristics. A weak influence of reproductive and menstrual factors on the familial risk emerged from the present study.

**Keywords:** breast cancer; familial risk; reproductive life factor; interactions

The association of a family history of breast cancer with an increased risk of breast cancer has been well documented. This risk increases with the number of affected relatives and a decreasing degree of kinship (Kelsey and Horm-Ross, 1993). Segregation analyses of large population-based family studies have shown that familial aggregation of breast cancer can be explained by the transmission of a dominant gene with a high lifetime penetrance (Williams and Anderson, 1984; Newman et al, 1988; Claus et al, 1991; Iselius et al, 1991). Linkage analyses of multiple breast and breast–ovarian cancer families led to the localization of two breast cancer genes accounting for a minority of cases (5–10%) (Hall et al; 1990; Narod et al, 1991; Wooster et al, 1994). However, more complex mechanisms have also been suggested, and several family studies have indicated a possible genetic heterogeneity for breast cancer (Demenais et al, 1986; Gilligan and Borecki, 1986; Andrieu et al, 1988; Goldstein et al, 1988; Goldstein and Amos, 1990).

Genetic factors do not explain all of the variation in breast cancer rates. In particular, several reproductive factors are well-established risk factors for breast cancer. These include an early age at menarche, a late age at menopause, a late age at first childbirth and nulliparity. The risks associated with other reproductive factors such as abortions, certain characteristics of the menstrual cycle, infertility and breast feeding are still controversial (Kelsey and Horm-Ross, 1993). Overall relative risks associated with reproductive factors are typically about 2.0 or less and even if oestrogen activity seems involved in breast cancer occurrence, the mechanisms underlying such effects are still obscure. In fact, except for the general observation that longer exposure to menstrual activity brings about an increased risk for breast cancer, no generally accepted mechanisms have been proposed to explain these epidemiological characteristics.

The difficulty in detecting relevant factors and understanding their role in the aetiology of breast cancer may be due to the use of inaccurate measures of oestrogen activity or to heterogeneity in susceptibility of the population of cases studied. For example, several studies have found that some reproductive factors might have a variable effect on the occurrence of breast cancer according to the existence or not of a family history of breast cancer (Adami et al, 1980; Bain et al, 1980; Brinton et al, 1982; Sattin et al, 1985; Olsson et al, 1985; Richardson et al, 1985; Dupont and Page, 1987; Negri et al, 1988; Malone and Daling, 1992; Parazzini et al, 1992; Sellers et al, 1992; 1993; Andrieu et al, 1993; 1995; Colditz et al, 1993; 1996). In a previous study we investigated the existence of an interaction between familial risk of breast cancer and abortion by combining six case–control studies from various

*Received 12 May 1997*
*Revised 9 September 1997*
*Accepted 1 October 1997*

*Correspondence to:* N Andrieu, Unité INSERM 351, Institut Gustave Roussy, 39 rue Camille Desmoulins, 94805 Villejuif Cedex, France

**Table 1** Studies included in the combined analysis

| Study | Country | Number of cases | Number of controls | Age at interview (years) | Year of interview |
|---|---|---|---|---|---|
| Rohan et al (1988) | Australia | 451 | 451 | 20–74 | 1982–1984 |
| Lee et al (1991) | Singapore | 200 | 420 | 24–88 | 1986–1988 |
| Lifanova et al (unpublished data) | Russia | 885 | 1068 | 21–87 | 1992–1994 |
| Richardson et al (1991) | France | 450 | 603 | 21–66 | 1983–1987 |
| Luporsi (1988) | France | 406 | 812 | 24–83 | 1985–1987 |
| Lê et al (1984) | France | 265 | 265 | 22–46 | 1982–1984 |
| Clavel et al (1991) | France | 495 | 785 | 20–56 | 1983–1987 |
| | Total | 3152 | 4404 | | |

countries. Our findings suggested a synergism between familial factors and abortion (Andrieu et al, 1995). In this paper, the combined analysis is extended to study the existence of an interaction between familial risk and other reproductive factors. In particular, the interaction between familial risk and breast cell mitotic activity (BCMA) as assessed by ovarian activity is investigated because of the potential importance of mitotic activity on genetically susceptible cells. The investigation combines the six case–control studies previously analysed and a seventh from Singapore. The aim of the present study was to investigate how the effect of family history is modified by reproductive factors. In addition, the risk associated with reproductive factors were examined in the two groups defined by the presence or absence of a family history of breast cancer.

### MATERIALS AND METHODS

The analysis included case–control studies from four countries, France, Australia, Russia and Singapore. The data sets were chosen because they had information on family history of breast cancer and as well as reproductive factors of interest, such as age at menarche, number of children, number of abortions, age at first childbirth, menopausal status and age at menopause. For all studies, family history of breast cancer was recalled by the subjects and was not verified from medical records. The present analysis included 3152 cases and 4404 controls. No family history, in this analysis, includes unknown family history. Most studies in the combined analysis have been published. The seven studies are briefly described in Table 1, and the main design features are presented below.

In a case–control study from South Australia (Rohan et al, 1988) the cases were obtained from the population-based South Australian Central Cancer Registry between 1982 and 1984 to investigate the relationship between dietary intake and the risk of breast cancer. Cases were between 20 and 74 years of age, with a histologically verified first diagnosis of breast cancer. For each case, one control was selected at random from the electoral roll from among women of approximately the same age as that of the case at diagnosis. Study subjects were interviewed in their homes by trained interviewers. In addition to information on usual dietary intake, information on family history of cancer in sisters, mother and grandmothers was recorded. For the present study, information about first-degree relatives only was provided (Rohan et al, 1988).

In a case–control study from Singapore (Lee et al, 1992), breast cancer patients were consecutive admissions to Singapore General Hospital and the National University Hospital between 1986 and 1988. About twice as many controls as cases were selected within 5-year age groups. Subjects were between 24 and 88 years of age. They were interviewed in hospital by experienced investigators. In addition to information on dietary intake, the interview also included questions about reproductive life factors and history of breast cancer in the subject's mother, sisters or maternal aunts.

Data were obtained from a case–control study performed in Moscow (Russia) (Lifanova et al, unpublished) that focused on diet, alcohol consumption and reproductive factors. Subjects were interviewed from 1992 to 1994. Cases were aged between 23 and 82 years old with histologically confirmed primary carcinoma of the breast and were recruited from four Moscow hospitals. Controls were women with minor non-chronic complaints registered in primary care polyclinics in Moscow. Information was recorded on the occurrence of breast cancer in the family (sisters, mother, aunts and grandmothers).

A case–control study carried out in Montpellier (France) (Richardson et al, 1991) focused on nutritional factors. Subjects were interviewed between 1983 and 1987. Cases were women aged between 26 and 66 years old with histologically confirmed primary carcinoma of the breast who were hospitalized in the Montpellier Cancer Institute and had not previously undergone any therapy. Controls were women of the same age range admitted for the first time into three different wards: neurology, neurosurgery and general surgery. These women were attending for a first diagnosis and hence were not being currently treated for chronic diseases. Information was recorded on the occurrence of breast cancer in the family (sisters, mother and aunts) and the number of sisters and aunts.

A case–control study carried out in Nancy Cancer Institute (France) between 1985 and 1987 (Luporsi, 1988) investigated the relationship between familial factors, alcohol, tobacco and obesity and the risk of breast cancer. Cases were between 24 and 83 years of age, with a histologically confirmed infiltrating breast carcinoma. Controls were women admitted into general surgery or general medicine wards. These women were examined to eliminate a diagnosis of cancer. Controls were matched to cases by age at interview (± 3 years), residential area and occupational status. Each case was matched to two controls. Information was recorded on the occurrence of breast cancer in the family (sisters, mother, aunts and grandmothers) and the number of sisters and aunts.

A multicentre case–control study performed in France between 1981 and 1984 (Lê et al, 1984) investigated the relationship between oral contraceptive use and the risk of breast cancer. Cases were between 20 and 45 years of age, with a histologically verified breast carcinoma diagnosed less than a year before the interview. Each case was matched with one control with respect to hospital, date of interview and age. This control was chosen from patients

 © Cancer Research Campaign 1998

with non-malignant diseases, excluding benign breast disease and severe or moderate cervical dysplasia. Information was recorded on the occurrence of breast cancer in the family (sisters, mother, aunts and grandmothers) and the number of sisters and aunts.

Data were obtained from a case–control study in five French hospitals between 1983 and 1987 (Clavel et al, 1991) that investigated the relationship between oral contraceptive use and the risk of breast cancer. Cases were between 20 and 56 years of age. They had a histologically confirmed infiltrating or in situ breast carcinoma. Three types of controls were eligible for each case: friends, colleagues or patients hospitalized for a non-malignant disease. The criteria for matching controls to cases were the centre, age at interview (± 5 years) and year of interview (± 14 months). Each case and her matching controls were interviewed by the same interviewer. The 111 controls with a malignant disease were excluded from the present analysis and the matching broken. Information was recorded on the occurrence of breast cancer in the family (sisters, mother, aunts and grandmothers) and the number of sisters and aunts.

### Statistical methods

In the first stage of the analysis, each study was analysed separately using unconditional logistic regression for the unmatched studies and conditional logistic regression for the matched studies. For each study, in order to examine interactions of family history with reproductive life variables, the risk of a family history of breast cancer was calculated separately in each stratum of the reproductive life factors. To test the interaction, a chi-square test for heterogeneity was performed by comparing the difference between the deviance of the above model and that of a model in which the familial risk was assumed to be the same in all strata. The risks of reproductive life factors were calculated for each subgroup: with or without family history of breast cancer. These analyses were performed using the software package EGRET.

Two different combined analyses were performed. The first one was performed using a classical approach in which the relative risks estimated using logistic regression were combined by taking a multivariate weighted average (WM). This method allows the point and interval estimates of relative risks to be obtained and provides tests of the effects on risk and of heterogeneity from study to study. Mathematical details are given elsewhere (Ewertz et al, 1990; Woolf, 1955). The interaction was tested as the statistical significance of the weighted average of the interaction terms using a 0.05 level of significance.

In the second analysis, the combined relative risks were estimated using a Bayesian random-effects model. A random-effects analysis assumes that the true effects in each study are not necessarily equal, but are random pertubations about some common mean effect. Inferences based on this model were obtained by the simulation technique known as Gibbs sampling (GS) (Gelfand and Smith, 1990). This model allows for between study heterogeneity in effects and hence gives wider 95% confidence intervals on the combined effects when heterogeneity is present.

The variables studied are age at menarche, age at first childbirth, number of children, age at menopause, and estimations of the duration of BCMA. Among nulliparous women, BCMA until first childbirth (pre-first-childbirth BCMA) was calculated by totalling the years between menarche and interview for premenopausal women or menopause for post-menopausal women. Among parous women, pre-first-childbirth BCMA was calculated as the years between menarche and first childbirth. The pre-first-childbirth BCMA was categorized into four classes [≤ 10 years, (11–15 years), (16–20 years), ≥ 21 years]. The lifetime BCMA was calculated by totalling the years of reproductive life between menarche and interview for premenopausal women, or menopause for post-menopausal women, and then subtracting the estimated total years of full-term pregnancy, which is the number of children multiplied by 0.75 (0.75 years = average length of pregnancy). Lifetime BCMA is equal to pre-first-childbirth BCMA in nulliparous women. Then lifetime BCMA was categorized in four classes [≤ 28 years, (28–32 years), (33–37 years), ≥ 38 years]. Because detailed information on incomplete pregnancies was not available for most studies, a precise assessment of BCMA was not possible. However, analyses were performed adjusting for total number of abortions (both spontaneous and induced) for six out of the seven studies (number of abortions not available for the Lee et al study).

### RESULTS

### Main effects of reproductive factors

First, the main effects of reproductive life factors were investigated. In three of the seven studies, a significant decrease in risk of breast cancer was found for women with an age at menarche of more than 15 years compared with those with an age at menarche less than 13 years (Richardson et al, Lê et al, Clavel et al) (data not shown). In the other four, although the odds ratio associated with an age at menarche of more than 15 years are not significantly different from unity, the point estimates were all less than one. The combined analysis confirmed this observation with an odds ratio of 0.75 (95% CI 0.65–0.87) with WM, and 0.73 (95% CI 0.61–0.87) with GS (Table 2). A significant trend of decreasing risk with increasing age at menarche is found in two of the studies (Clavel et al, Lê et al) and in the WM combined analysis.

In all the studies, a decrease in risk of breast cancer was found for women with three or more children. However, in only two studies is this risk significantly different from unity (Lee et al, Clavel et al). The two methods for combining data lead to identical point estimates of odds ratios: 0.89 (95% CI 0.77–1.03 with WM, 95% CI 0.76–1.05 with GS) associated with one or two children, 0.71 (95% CI 0.60–0.83 with WM, 95% CI 0.60–0.86 with GS) associated with three or more children (Table 2). A trend of decreasing risk with increasing number of children is significant in three of the studies (Lee et al, Luporsi, Clavel et al) and in the WM combined analysis.

In only two out of the seven studies was a significantly decreased risk of breast cancer found for women aged 24 years or less at first childbirth (Lee et al, Lifanova et al) compared with nulliparous women. In five out of the seven studies, an increased risk of breast cancer was observed with increasing age at first child among parous women. In only three studies (Lee et al, Lifanova et al, Luporsi) is the trend found to be significant (data not shown). The combined analyses confirmed a significantly decreased risk of breast cancer with an age at first childbirth of less than 24 years [0.81 (95% CI 0.69–0.95) with WM, 0.80 (95% CI 0.67–0.96) with GS] and an increasing risk of breast cancer with increasing age at first childbirth among parous women (with WM, *P* trend = 0.002) (Table 2).

© Cancer Research Campaign 1998

**Table 2** Combined odds ratios of breast cancer associated with age at menarche, age at first childbirth, number of children and age at menopause

| Reproductive factors | OR | 95 %CI | $P_t$ |
|---|---|---|---|
| Age at menarche | | | |
| Fixed effect (Woolf) | | | |
| ≤ 12 years | 1[a] | | |
| (13–14) | 0.96 | (0.86–1.08) | |
| ≥ 15 years | 0.75 | (0.65–0.87) | < 0.001 |
| Random effect (Gibbs sampling) | | | |
| ≤ 12 years | 1[a] | | |
| (13–14) | 0.97 | (0.83–1.13) | |
| ≥ 15 years | 0.73 | (0.61–0.87) | |
| Age at first child | | | |
| Fixed effect (Woolf) | | | |
| No childbirth | 1[b] | | |
| ≤ 24 years | 0.81 | (0.69–0.95) | |
| (25–29) | 0.92 | (0.78–1.09) | 0.002 |
| ≥ 30 years | 1.10 | (0.90–1.34) | |
| Random effect (Gibbs sampling) | | | |
| No childbirth | 1[b] | | |
| ≤ 24 years | 0.84 | (0.72–1.00) | |
| (25–29) | 0.96 | (0.81–1.14) | |
| ≥ 30 years | 1.10 | (0.89–1.35) | |
| Number of children | | | |
| Fixed effect (Woolf) | | | |
| no child | 1[c] | | |
| 1–2 | 0.89 | (0.77–1.03) | |
| ≥ 3 | 0.71 | (0.60–0.83) | < 0.001 |
| Random effect (Gibbs sampling) | | | |
| No child | 1[c] | | |
| 1–2 | 0.89 | (0.76–1.05) | |
| ≥ 3 | 0.71 | (0.60–0.86) | |
| Age at menopause | | | |
| Fixed effect (Woolf) | | | |
| Premenopausal | 1[d] | | |
| Menopausal < 50 years | 0.62 | (0.52–0.74) | |
| Menopausal ≥ 50 years | 0.93[e] | (0.93–1.12) | |
| Random effect (Gibbs sampling) | | | |
| Premenopausal | 1[d] | | |
| Menopausal < 50 years | 0.61 | (0.48–0.78) | |
| Menopausal ≥ 50 years | 0.92 | (0.70–1.21) | |

[a]Adjusted for age at interview, age at first child, number of abortions (except for Lee et al), number of children, menopausal status and family history of breast cancer; [b]adjusted for age at interview, number of abortions (except for Lee et al), age at menarche, number of children, menopausal status and family history of breast cancer; [c]adjusted for age at interview, age at first child, number of abortions (except for Lee et al), age at menarche, menopausal status and family history of breast cancer; [d]adjusted for age at interview, age at first child, number of abortions (except for Lee et al), number of children, age at menarche and family history of breast cancer; [e]odds ratio estimated on six data sets (Lê et al excluded).

In all the studies, a decreased risk of breast cancer was found associated with menopause, especially when menopause occurred before 50 years of age (data not shown). However, being menopausal before 50 years of age is associated with a significant decreased risk of breast cancer in only four of the studies (Richardson et al, Luporsi, Lê et al, Clavel et al). The two methods for combining data lead to similar point estimates of odds ratios for women who were menopausal before 50 years of age: 0.62 (95% CI 0.52–0.74) with WM, and 0.61 (95% CI 0.48–0.78) with GS, and for women who were menopausal after 50 years of age (OR = 0.93 with WM; OR = 0.92 with GS) (Table 2). Effects of artificial and natural menopause are very similar and lie between the point estimates for menopause at age less than 50 and menopause at age greater than 50 (data not shown).

The main effect of pre-first-childbirth BCMA is shown in Table 3. In only two of the seven studies, a clear trend of increased risk of breast cancer with increasing duration of pre-first-childbirth BCMA was found (Lee et al, Lifanova et al). Nevertheless, combined analyses led to a significant increased risk of breast cancer associated with an increased duration of pre-first-childbirth BCMA (*P* trend in WM is less than 0.001).

In all studies, an increased risk of breast cancer was associated with an increased lifetime BCMA (Table 4). Moreover, an increasing trend is significant ($P < 0.05$) in five of the studies (Lee et al, Lifanova et al, Richardson et al, Luporsi, Clavel et al) and in the combined analysis.

For both pre-first-childbirth BCMA and lifetime BCMA, confidence intervals and point estimates of odds ratios from the two methods for combined analyses are similar.

© Cancer Research Campaign 1998

**Table 3** Risk of breast cancer associated with duration of pre-first-childbirth BCMA

| Study | OR[a] | 95% CI | $P_t$ |
|---|---|---|---|
| Rohan et al (1988) | | | |
| ≤ 10 years | 1 | | |
| (11–15) | 1.10 | (0.80–1.51) | |
| (16–20) | 0.94 | (0.61–1.45) | n.s. |
| ≥ 21 years | 1.11 | (0.71–1.73) | |
| Lee et al (1991) | | | |
| ≤ 10 years | 1 | | |
| (11–15) | 1.52 | (0.97–2.37) | |
| (16–20) | 1.54 | (0.87–2.74) | < 0.001 |
| ≥ 21 years | 7.16 | (3.27–15.7) | |
| Lifanova et al (unpublished data) | | | |
| ≤ 10 years | 1 | | |
| (11–15) | 1.50 | (1.17–1.94) | |
| (16–20) | 1.68 | (1.18–2.38) | < 0.001 |
| ≥ 21 years | 1.72 | (1.25–2.37) | |
| Richardson et al (1991) | | | |
| ≤ 10 years | 1 | | |
| (11–15) | 1.51 | (1.12–2.05) | |
| (16–20) | 0.89 | (0.56–1.42) | 0.034 |
| ≥ 21 years | 1.19 | (0.79–1.77) | |
| Luporsi (1988) | | | |
| ≤ 10 years | 1 | | |
| (11–15) | 0.99 | (0.74–1.34) | |
| (16–20) | 1.69 | (1.09–2.62) | 0.087 |
| ≥ 21 years | 1.08 | (0.73–1.59) | |
| Lê et al (1984) | | | |
| ≤ 10 years | 1 | | |
| (11–15) | 1.00 | (0.63–1.58) | |
| (16–20) | 0.89 | (0.49–1.61) | n.s. |
| ≥ 21 years | 1.22 | (0.68–2.20) | |
| Clavel et al (1991) | | | |
| ≤ 10 years | 1 | | |
| (11–15) | 1.17 | (0.89–1.53) | |
| (16–20) | 1.86 | (1.26–2.75) | 0.019 |
| ≥ 21 years | 1.30 | (0.89–1.89) | |
| Combined analysis | | | |
| Fixed effect (Woolf) | | | |
| ≤ 10 years | 1 | | |
| (11–15) | 1.25 | (1.11–1.40) | |
| (16–20) | 1.35 | (1.14–1.60) | < 0.001 |
| ≥ 21 years | 1.40 | (1.20–1.65) | |
| Random effect (Gibbs sampling) | | | |
| ≤ 10 years | 1 | | |
| (11–15) | 1.23 | (1.03–1.50) | |
| (16–20) | 1.33 | (1.06–1.66) | |
| ≥ 21 years | 1.45 | (1.17–1.82) | |

[a]Adjusted for age at interview, number of abortions (except for Lee et al), number of children, menopausal status and family history of breast cancer; $P_t$, $P$-value for trend.

### Variation of familial risk according to reproductive factors

The main effect of family history was investigated. The effect was significant in all studies [Rohan et al, 1.6 (95% CI 1.0–2.7); Lee et al, 3.1 (95% CI 1.3–7.2); Lifanova et al, 4.1 (95% CI 2.6–6.7); Richardson et al, 2.8 (95% CI 1.7–4.6); Luporsi, 2.7 (95% CI 1.9–3.9); Lê et al, 1.9 (95% CI 1.2–3.0); Clavel et al, 1.5 (95% CI 1.1–2.0)]. The odds ratio associated with a family history estimated from the combined analysis was 2.2 (95% CI 1.9–2.6).

In Tables 5–7 results for the effect of reproductive life factors stratified by family history of breast cancer and for the effect of a family history stratified by different levels of the reproductive life factors are presented. These tables can be read in two different ways, depending on whether one is interested in the modifications of the familial risk because of reproductive life factors or by the modifications of the risk from reproductive life factors because of a familial factor. None of the tests of heterogeneity of effects between studies were statistically significant; nor were any of the tests for interaction of family history with reproductive life variables statistically significant, either within individual studies or in the combined analyses.

© Cancer Research Campaign 1998 

**Table 4** Risk of breast cancer associated with duration of lifetime BCMA

| Study | OR[a] | 95% CI | $P_t$ |
|---|---|---|---|
| Rohan et al (1988) | | | |
| ≤ 27 years | 1 | | |
| (28–32) | 0.99 | (0.67–1.48) | |
| (33–37) | 1.28 | (0.87–1.89) | 0.106 |
| ≤ 38 years | 1.61 | (1.02–2.56) | |
| Lee et al (1991) | | | |
| ≤ 27 years | 1 | | |
| (28–32) | 1.58 | (0.99–2.51) | |
| (33–37) | 2.44 | (1.44–4.16) | 0.012 |
| ≥ 38 years | 1.97 | (0.89–4.35) | |
| Lifanova et al (unpublished data) | | | |
| ≤ 27 years | 1 | | |
| (28–32) | 1.30 | (0.93–1.83) | |
| (33–37) | 1.85 | (1.32–2.60) | < 0.001 |
| ≥ 38 years | 2.25 | (1.48–3.43) | |
| Richardson et al (1991) | | | |
| ≤ 27 years | 1 | | |
| (28–32) | 1.59 | (1.07–2.37) | |
| (33–37) | 2.44 | (1.63–3.64) | < 0.001 |
| ≥ 38 years | 2.80 | (1.72–4.56) | |
| Luporsi (1988) | | | |
| ≤ 27 years | 1 | | |
| (28–32) | 1.52 | (0.94–2.45) | |
| (33–37) | 2.20 | (1.33–3.64) | < 0.001 |
| ≥ 38 years | 2.36 | (1.36–4.12) | |
| Lê et al (1984) | | | |
| ≤ 27 years | 1 | | |
| (28–32) | 1.28 | (0.68–2.38) | |
| (33–37) | 1.69 | (0.43–6.56) | n.s. |
| ≥ 38 years | | | |
| Clavel et al (1991) | | | |
| ≤ 27 years | 1 | | |
| (28–32) | 1.64 | (1.03–2.61) | |
| (33–37) | 2.57 | (1.59–4.17) | < 0.001 |
| ≥ 38 years | 2.93 | (1.74–4.93) | |
| Combined analysis | | | |
| Fixed effect (Woolf) | | | |
| ≤ 27 years | 1 | | |
| (28–32) | 1.40 | (1.20–1.63) | |
| (33–37) | 2.01 | (1.71–2.38) | < 0.001 |
| ≥ 38 years | 2.38 | (1.93–2.94) | |
| Random effect (Gibbs sampling) | | | |
| ≤ 27 years | 1 | | |
| (28–32) | 1.30 | (1.12–1.51) | |
| (33–37) | 1.85 | (1.58–2.18) | |
| ≥ 38 years | 2.16 | (1.77–2.65) | |

[a]Adjusted for age at interview, number of abortions (except for Lee et al), age at first child and family history of breast cancer.

The point estimate of the odds ratio associated with a family history of breast cancer is higher for women who were older than 15 years at menarche than for women who were younger than 13 years in four of the seven studies and is lower in the three others (data not shown). For both methods, the combined analyses illustrate this discrepancy leading to similar odds ratios whatever the age at menarche (Table 5).

The point estimate of the familial risk odds ratio ($OR_{FR}$) increases as age at first childbirth increases among parous women in five of the seven studies (Lifanova et al, Richardson et al, Luporsi, Lê et al, Clavel et al) (data not shown). Combined analyses confirmed these observations with an odds ratio of 1.95 for women who were less than 25 years old at their first childbirth, 2.40 for those who were between 25 and 29 years old and 2.80 for those who were older than 25 years, using GS. The pattern of $OR_{FR}$ is less clear using WM (Table 6).

In six of the seven studies, the point estimates of $OR_{FR}$ are higher for women having three or more children than for those having one or two (Rohan et al, Lee et al, Lifanova et al, Richardson et al, Luporsi, Clavel et al). Among these six studies, the $OR_{FR}$ point estimates are higher for women who have three or more children compared with nulliparous women in only three of

© Cancer Research Campaign 1998

**Table 5** Variation of breast cancer risk associated with the age at menarche, age at first childbirth, number of children, age at menopause according to the presence or not of a family history of breast cancer and variation of familial risk according to the age at menarche, age at first childbirth, number of children, age at menopause: results of combined analysis.

| | Without family history of breast cancer | | | | With family history of breast cancer | | | | Familial risk | | |
|---|---|---|---|---|---|---|---|---|---|---|---|
| **Reproductive factors** | **Cases** | **Controls** | **OR** | **95% CI** | **Cases** | **Controls** | **OR** | **95% CI** | **OR** | **95% CI** | **P[a]** |
| Age at menarche[b] | | | | | | | | | | | |
| Fixed effect (Woolf) | | | | | | | | | | | |
| ≤ 12 years | 832 | 1172 | 1 | | 150 | 100 | 1 | | 2.22 | (1.67–2.94) | |
| (13–14) | 1222 | 1795 | 0.98 | (0.87–1.10) | 195 | 142 | 0.91 | (0.60–1.39) | 2.07 | (1.13–3.81) | n.s. |
| ≥ 15 years | 465 | 872 | 0.76 | (0.65–0.88) | 75 | 67 | 0.76 | (0.44–1.29) | 2.22 | (1.13–4.34) | |
| Unknown | 9 | 22 | – | – | 0 | 0 | – | – | – | – | |
| *Random effect (Gibbs sampling)* | | | | | | | | | | | |
| ≤ 12 years | 832 | 1172 | 1 | | 150 | 100 | 1 | | 2.29 | (1.61–3.31) | |
| (13–14) | 1222 | 1795 | 0.98 | (0.83–1.16) | 195 | 142 | 0.93 | (0.65–1.35) | 2.18 | (1.60–3.01) | |
| ≥ 15 years | 465 | 872 | 0.73 | (0.59–0.87) | 75 | 67 | 0.75 | (0.47–1.20) | 2.25 | (1.47–3.41) | |
| Age at first childbirth[c] | | | | | | | | | | | |
| Fixed effect (Woolf) | | | | | | | | | | | |
| No childbirth | 390 | 514 | 1 | | 60 | 32 | 1 | | 2.18[d] | (1.33–3.59) | |
| ≤ 24 years | 1155 | 1969 | 0.86[d] | (0.73–1.02) | 181 | 165 | 0.69[d] | (0.38–1.28) | 1.76[d] | (0.64–4.85) | |
| (25–29) | 669 | 995 | 0.94[d] | (0.79–1.11) | 124 | 87 | 1.18[d] | (0.55–2.53) . | 2.74[d] | (0.90–8.35) | n.s. |
| ≥ 30 years | 309 | 368 | 1.10[e] | (0.85–1.42) | 55 | 24 | 1.07[e] | (0.45–2.55) | 1.86[e] | (0.58–6.02) | |
| Unknown | 5 | 14 | – | – | 0 | 1 | – | – | – | – | |
| Random effect (Gibbs Sampling) | | | | | | | | | | | |
| No childbirth | 390 | 514 | 1 | | | | 1 | | 2.74 | (1.64–4.75) | |
| ≤ 24 years | 1155 | 1969 | 0.83 | (0.69–1.00) | 60 | 32 | 0.63 | (0.38–1.02) | 1.95 | (1.41–2.72) | |
| (25–29) | 669 | 995 | 0.91 | (0.76–1.09) | 181 | 165 | 0.83 | (0.49–1.36) | 2.40 | (1.66–3.60) | |
| ≥ 30 years | 309 | 368 | 1.09 | (0.87–1.35) | 55 | 87 | 1.17 | (0.60–2.27) | 2.80 | (1.61–5.08) | |
| Number of children[f] | | | | | | | | | | | |
| Fixed effect (Woolf) | | | | | | | | | | | |
| 0 | 390 | 515 | 1 | | 60 | 32 | 1 | | 2.35[d] | (1.44–3.84) | |
| (1–2) | 1431 | 2011 | 0.94[d] | (0.80–1.11) | 235 | 174 | 0.78[d] | (0.47–1.32) | 1.96[d] | (0.95–4.06) | 0.17 |
| ≥ 3 | 707 | 1330 | 0.77[g] | (0.63–0.94) | 125 | 103 | 0.76[g] | (0.43–1.33) | 2.35[g] | (1.69–3.26) | |
| Unknown | 0 | 5 | – | – | 0 | 0 | – | – | – | – | |
| Random effect (Gibbs Sampling) | | | | | | | | | | | |
| 0 | 390 | 515 | 1 | | 60 | 32 | 1 | | 2.72 | (1.67–4.67) | |
| (1–2) | 1431 | 2011 | 0.93 | (0.77–1.12) | 235 | 174 | 0.69 | (0.42–1.13) | 2.01 | (1.50–2.67) | |
| ≥ 3 | 707 | 1330 | 0.73 | (0.60–0.91) | 125 | 103 | 0.65 | (0.38–1.11) | 2.38 | (1.66–3.34) | |
| Age at menopause[h] | | | | | | | | | | | |
| Fixed effect (Woolf) | | | | | | | | | | | |
| Premenopausal | 1206 | 1811 | 1 | | 237 | 165 | 1 | | 2.17 | (1.84–2.57) | |
| < 50 years | 576 | 1135 | 0.62 | (0.52–0.74) | 88 | 85 | 0.62 | (0.42–0.91) | 2.16 | (1.55–3.01) | n.s. |
| ≥ 50 years | 735 | 900 | 0.94[i] | (0.77–1.14) | 95 | 59 | 0.87[i] | (0.55–1.38) | 2.11[i] | (1.43–3.14) | |
| Random effect (Gibbs sampling) | | | | | | | | | | | |
| Premenopausal | 1206 | 1811 | 1 | | 237 | 165 | 1 | | 2.41 | (1.72–3.64) | |
| < 50 years | 576 | 1135 | 0.62 | (0.50–0.76) | 88 | 85 | 0.63 | (0.41–0.95) | 2.27 | (1.45–3.58) | |
| ≥ 50 years | 735 | 900 | 0.94 | (0.73–1.16) | 95 | 59 | 0.91 | (0.58–1.42) | 2.07 | (1.30–3.32) | |

[a]Test for interaction; [b]adjusted for age at interview, age at first child, number of abortions (except for Lee et al), number of children, menopausal status; [c]adjusted for age at interview, age at menarche, number of abortions (except for Lee et al), number of children, menopausal status; [d]odds ratios estimated on six data sets (Lee et al excluded); [e]odds ratios estimated on five data sets (Richardson et al and Lee et al excluded); [f]adjusted for age at interview, age at menarche, age at first child, number of abortions (except for Lee et al), menopausal status; [g]odds ratios estimated on five data sets (Lifanova et al and Lee et al excluded); [h]adjusted for age at interview, age at menarche, number of abortions (except for Lee et al), number of children, age at first child; [i]odds ratios estimated on six data sets (Lê et al excluded).

the six (Lifanova et al, Luporsi, Clavel et al) (data not shown). Combined analyses led to similar observations with a $OR_{FR}$ of 1.96 with WM and 2.01 with GS for women with one or two children and of 2.35 with WM and 2.38 with GS for women with three or more children (Table 5).

The pattern of variation in familial risk according to menopausal status and age at menopause differs from study to study. A significant interaction is found in Lifanova and colleague's study ($P$ = 0.04) (data not shown). However, the combined analyses show a decrease in familial risk from premenopausal women, to women menopausal before 50 years old, to women menopausal after 50 years old. This decreasing risk is more noticeable using GS with an odds ratio of 2.41 for premenopausal women, 2.27 for women menopausal before 50 years old and 2.07 for women menopausal after 50 years old (Table 5).

The variation of the familial risk according to pre-first-childbirth BCMA differs from study to study and subsequently the combined analyses lead to an unclear pattern of the familial risk (Table 6).

The pattern of variation in familial risk according to lifetime BCMA differs from study to study (Table 7). However in five of the seven studies, the point estimates of the odds ratio associated

© Cancer Research Campaign 1998 

**Table 6** Variation of breast cancer risk and of familial risk associated with duration of pre-first-childbirth BCMA according to the presence or not of a family history of breast cancer and variation of familial risk according to duration of pre-first childbirth BCMA

| | Without family history of breast cancer | | | | With family history of breast cancer | | | | familial risk | | |
|---|---|---|---|---|---|---|---|---|---|---|---|
| Study | Cases | Controls | OR[a] | 95% CI | Cases | Controls | OR[a] | 95% CI | OR[a] | 95% CI | P[b] |
| Rohan et al (1988) | | | | | | | | | | | |
| ≤ 10 years | 153 | 167 | 1 | | 13 | 10 | 1 | | 1.50 | (0.64–3.55) | |
| (11–15) | 131 | 135 | 1.09 | (0.79–1.52) | 15 | 10 | 1.12 | (0.35–3.56) | 1.54 | (0.66–3.60) | |
| (16–20) | 52 | 57 | 0.94 | (0.60–1.48) | 5 | 4 | 0.92 | (0.19–4.35) | 1.46 | (0.37–5.77) | n.s. |
| ≥ 21 years | 66 | 61 | 1.08 | (0.68–1.70) | 8 | 3 | 1.71 | (0.35–8.30) | 2.39 | (0.60–9.48) | |
| Unknown | 8 | 4 | – | – | 0 | 0 | – | – | – | – | |
| Lee et al (1991) | | | | | | | | | | | |
| ≤ 10 years | 77 | 233 | 1 | | 2 | 4 | 1 | | 1.82 | (0.32–10.3) | |
| (11–15) | 54 | 103 | 1.54 | (0.98–2.41) | 3 | 4 | 1.29 | (0.13–12.82) | 1.52 | (0.32–7.14) | |
| (16–20) | 26 | 52 } | 1.44 | (0.79–2.61) | 5 | 2 } | 4.06 | (0.37–43.98) | 5.12 | (0.93–28.3) | n.s. |
| ≥ 21 years | 27 | 14 } | | | 4 | 0 } | | | | | |
| Unknown | 2 | 8 | – | – | 0 | 0 | – | – | – | – | |
| Lifanova et al (unpublished data) | | | | | | | | | | | |
| ≤ 10 years | 232 | 398 | 1 | | 25 | 11 | 1 | | 4.00 | (1.92–8.30) | |
| (11–15) | 180 | 211 | 1.51 | (1.16–1.96) | 28 | 9 | 1.44 | (0.51–4.06) | 3.82 | (1.75–8.33) | |
| (16–20) | 76 | 86 | 1.65 | (1.15–2.36) | 9 | 1 | 3.07 | (0.34–27.8) | 7.43 | (0.90–61.2) | n.s. |
| ≥ 21 years | 119 | 115 | 1.72 | (1.24–2.39) | 12 | 3 | 1.69 | (0.39–7.26) | 3.91 | (1.07–14.3) | |
| Richardson et al (1991) | | | | | | | | | | | |
| ≤ 10 years | 171 | 283 | 1 | | 21 | 20 | 1 | | 1.79 | (0.93–3.42) | |
| (11–15) | 125 | 138 | 1.43 | (1.04–1.95) | 16 | 5 | 3.09 | (0.93–10.2) | 3.86 | (1.35–11.1) | |
| (16–20) | 33 | 59 | 0.79 | (0.49–1.28) | 6 | 1 | 5.45 | (0.59–50.1) | 12.30 | (1.40–108) | n.s. |
| ≥ 21 years | 66 | 82 | 1.11 | (0.73–1.67) | 7 | 2 | 3.06 | (0.56–16.8) | 4.90 | (0.98–24.8) | |
| Unknown | 5 | 12 | – | – | 0 | 1 | – | – | – | – | |
| Luporsi (1988) | | | | | | | | | | | |
| ≤ 10 years | 144 | 362 | 1 | | 31 | 31 | 1 | | 2.68 | (1.56–4.61) | |
| (11–15) | 90 | 209 | 1.04 | (0.75–1.43) | 25 | 27 | 0.80 | (0.38–1.70) | 2.07 | (1.13–3.79) | |
| (16–20) | 37 | 57 | 1.55 | (0.94–2.41) | 11 | 2 | 5.17 | (1.05–25.5) | 9.23 | (1.92–44.3) | n.s. |
| ≥ 21 years | 56 | 114 | 1.09 | (0.72–1.64) | 12 | 10 | 1.00 | (0.37–2.72) | 2.48 | (1.00–6.14) | |
| Lê et al (1984) | | | | | | | | | | | |
| ≤ 10 years | 90 | 102 | 1 | | 26 | 19 | 1 | | 1.63 | (0.83–3.21) | |
| (11–15) | 54 | 64 | 0.95 | (0.58–1.57) | 20 | 11 | 1.24 | (0.47–3.30) | 2.13 | (0.92–4.91) | |
| (16–20) | 24 | 31 | 0.84 | (0.45–1.60) | 6 | 4 | 1.13 | (0.29–4.48) | 2.19 | (0.53–8.95) | n.s. |
| ≥ 21 years | 34 | 30 | 1.16 | (0.62–2.17) | 11 | 4 | 1.52 | (0.40–5.76) | 2.15 | (0.63–7.30) | |
| Clavel et al (1991) | | | | | | | | | | | |
| ≤ 10 years | 144 | 301 | 1 | | 34 | 41 | 1 | | 1.79 | (1.08–2.96) | |
| (11–15) | 132 | 230 | 1.19 | (0.88–1.60) | 40 | 44 | 1.05 | (0.56–1.97) | 1.58 | (0.97–2.56) | |
| (16–20) | 57 | 58 | 2.00 | (1.31–3.08) | 14 | 12 | 1.30 | (0.53–9.23) | 1.16 | (0.49–2.75) | n.s. |
| ≥ 21 years | 63 | 85 | 1.41 | (0.94–2.11) | 11 | 14 | 0.81 | (0.32–2.06) | 1.03 | (0.44–2.44) | |
| Combined analysis | | | | | | | | | | | |
| Fixed effect (Woolf) | | | | | | | | | | | |
| ≤ 10 years | 1011 | 1846 | 1 | | 152 | 136 | 1 | | 2.14 | (1.66–2.75) | |
| (11–15) | 766 | 1090 | 1.26 | (1.11–1.42) | 147 | 110 | 1.17 | (0.82–1.65) | 1.98 | (1.49–2.64) | n.s. |
| (16–20) | 305 | 400 | 1.32 | (1.10–1.57) | 56 | 26 | 1.62 | (0.94–2.78) | 2.62 | (1.56–4.41) | |
| ≥ 21 years | 431 | 501 | 1.32[c] | (1.11–1.56) | 65 | 36 | 1.32[c] | (0.80–2.19) | 2.11[c] | (1.34–3.33) | |
| Unknown | 15 | 24 | – | | 0 | 1 | – | – | – | – | |
| Random effect (Gibbs sampling) | | | | | | | | | | | |
| ≤ 10 years | 1011 | 1846 | 1 | | 152 | 136 | 1 | | 2.09 | (1.55–2.81) | |
| (11–15) | 766 | 1090 | 1.25 | (1.05–1.47) | 147 | 110 | 1.22 | (0.85–1.76) | 2.05 | (1.51–2.83) | |
| (16–20) | 305 | 400 | 1.30 | (1.05–1.60) | 56 | 26 | 1.90 | (1.13–3.30) | 3.03 | (1.82–5.20) | |
| ≥ 21 years | 431 | 501 | 1.44 | (1.18–1.79) | 65 | 36 | 1.53 | (0.95–2.51) | 2.27 | (1.44–3.71) | |

[a]Adjusted for age at interview, number of abortions (except for Lee et al), number of children, menopausal status; [b]test for interaction; [c]odds ratios estimated on six data sets (Lee et al excluded).

© Cancer Research Campaign 1998

**Table 7** Variation of breast cancer risk associated with duration of lifetime BCMA according to the presence or not of a family history of breast cancer and variation of familial risk according to duration of lifetime BCMA

| | Without family history of breast cancer | | | | With family history of breast cancer | | | | Familial risk | | |
|---|---|---|---|---|---|---|---|---|---|---|---|
| Study | Cases | Controls | OR[a] | 95% CI | Cases | Controls | OR[a] | 95% CI | OR[a] | 95% CI | *P*[b] |
| Rohan et al (1988) | | | | | | | | | | | |
| ≤ 27 years | 99 | 111 | 1 | | 9 | 8 | 1 | | 1.32 | (0.49–3.58) | |
| (28–32) | 85 | 109 | 0.96 | (0.63–1.45) | 10 | 6 | 1.57 | (0.39–6.37) | 2.16 | (0.75–6.22) | |
| (33–37) | 134 | 133 | 1.28 | (0.85–1.91) | 13 | 9 | 1.38 | (0.38–4.98) | 1.42 | (0.59–3.45) | n.s. |
| ≥ 38 years | 78 | 64 | 1.58 | (0.98–2.55) | 9 | 4 | 2.12 | (0.46–9.81) | 1.77 | (0.52–6.05) | |
| Unknown | 14 | 7 | – | – | 0 | 0 | – | – | – | – | |
| Lee et al (1991) | | | | | | | | | | | |
| ≤ 27 years | 54 | 162 | 1 | | 4 | 5 | 1 | | 2.06 | (0.52–8.23) | |
| (28–32) | 61 | 133 | 1.56 | (0.98–2.49) | 4 | 2 | 2.85 | (0.31–25.60) | 3.76 | (0.65–21.7) | |
| (33–37) | 51 | 85 | } 2.30 | (1.39–3.83) | 6 | 2 | } 3.46 | (0.49–24.50) | 3.10 | (0.74–13.1) | n.s. |
| ≥ 38 years | 15 | 24 | | | 0 | 1 | | | | | |
| Unknown | 2 | 6 | – | – | 0 | 0 | – | – | – | – | |
| Lifanova et al (unpublished data) | | | | | | | | | | | |
| ≤ 27 years | 85 | 188 | 1 | | 14 | 5 | 1 | | 6.30 | (2.19–18.1) | |
| (28–32) | 144 | 235 | 1.32 | (0.93–1.87) | 21 | 7 | 1.06 | (0.28–4.08) | 5.07 | (2.10–12.3) | |
| (33–37) | 268 | 289 | 1.92 | (1.36–2.72) | 27 | 9 | 0.95 | (0.26–3.45) | 3.12 | (1.44–6.80) | n.s. |
| ≥ 38 years | 110 | 95 | 2.33 | (1.52–3.58) | 12 | 3 | 1.20 | (0.23–6.28) | 3.24 | (0.87–12.0) | |
| Unknown | 0 | 3 | – | – | 0 | 0 | – | – | – | – | |
| Richardson et al (1991) | | | | | | | | | | | |
| ≤ 27 years | 69 | 180 | 1 | | 14 | 14 | 1 | | 2.71 | (1.22–6.01) | |
| (28–32) | 88 | 135 | 1.65 | (1.09–2.50) | 9 | 10 | 0.97 | (0.29–3.19) | 1.60 | (0.61–4.19) | |
| (33–37) | 149 | 158 | 2.36 | (1.56–3.59) | 14 | 3 | 4.45 | (1.03–19.2) | 5.11 | (1.44–18.2) | n.s. |
| ≥ 38 years | 92 | 83 | 2.67 | (1.61–4.43) | 13 | 2 | 6.08 | (1.13–32.8) | 6.17 | (1.35–28.3) | |
| Unknown | 2 | 18 | – | – | 0 | 0 | – | – | – | – | |
| Luporsi (1988) | | | | | | | | | | | |
| ≤ 27 years | 57 | 195 | 1 | | 16 | 15 | 1 | | 3.57 | (1.58–8.06) | |
| (28–32) | 66 | 155 | 1.84 | (1.10–3.00) | 14 | 24 | 0.84 | (0.29–2.42) | 1.63 | (0.77–3.46) | |
| (33–37) | 127 | 250 | 2.52 | (1.53–4.15) | 32 | 20 | 2.52 | (0.89–7.11) | 3.57 | (1.84–6.92) | n.s. |
| ≥ 38 years | 77 | 142 | 3.01 | (1.75–5.18) | 17 | 11 | 2.10 | (0.66–6.67) | 2.49 | (1.07–5.76) | |
| Lê et al (1984) | | | | | | | | | | | |
| ≤ 27 years | 136 | 156 | 1 | | 40 | 26 | 1 | | 1.85 | (1.06–3.21) | |
| (28–32) | 60 | 66 | } 1.44 | (0.65–3.19) | 23 | 12 | } 1.60 | (0.57–4.46) | 2.60 | (1.08–6.23) | n.s. |
| (33–37) | 6 | 5 | | | 0 | 0 | | | | | |
| ≥ 38 years | 0 | 0 | – | – | 0 | 0 | – | – | – | – | |
| Clavel et al (1991) | | | | | | | | | | | |
| ≤ 27 years | 138 | 288 | 1 | | 36 | 44 | 1 | | 1.18 | (0.70–1.87) | |
| (28–32) | 97 | 185 | 1.46 | (1.01–2.10) | 42 | 43 | 2.08 | (1.01–4.27) | 1.68 | (0.92–3.05) | |
| (33–37) | 132 | 170 | 2.05 | (1.32–3.19) | 11 | 13 | 3.86 | (1.77–8.42) | 2.21 | (1.18–4.14) | n.s. |
| ≥ 38 years | 29 | 31 | 2.94 | (1.51–5.72) | 11 | 11 | 3.13 | (1.07–9.10) | 1.25 | (0.45–3.50) | |
| Combined analysis | | | | | | | | | | | |
| Fixed effect (Woolf) | | | | | | | | | | | |
| ≤ 27 years | 639 | 1268 | 1 | | 132 | 130 | 1 | | 1.89 | (1.44–2.48) | |
| (28–32) | 617 | 1035 | 1.38 | (1.18–1.62) | 106 | 87 | 1.58 | (1.05–2.37) | 2.16 | (1.57–2.96) | n.s. |
| (33–37) | 852 | 1084 | 1.94[c] | (1.63–2.31) | 121 | 62 | 2.79[c] | (1.78–4.38) | 2.72[c] | (2.00–3.91) | |
| ≥ 38 years | 402 | 441 | 2.14[d] | (1.72–2.66) | 61 | 30 | 2.53[d] | (1.44–4.44) | 2.21[d] | (1.36–3.59) | |
| Unknown | 18 | 33 | – | – | 0 | 0 | – | – | – | – | |
| Random effect (Gibbs sampling) | | | | | | | | | | | |
| ≤ 27 years | 639 | 1268 | 1 | | 132 | 130 | 1 | | 2.03 | (1.50–2.88) | |
| (28–32) | 617 | 1035 | 1.29 | (1.10–1.50) | 106 | 87 | 1.39 | (0.94–2.04) | 2.12 | (1.51–3.04) | |
| (33–37) | 852 | 1084 | 1.80 | (1.52–2.11) | 121 | 62 | 2.48 | (1.63–3.71) | 2.71 | (1.84–4.03) | |
| ≥38 years | 402 | 441 | 2.12 | (1.72–2.60) | 61 | 30 | 2.57 | (1.56–4.36) | 2.38 | (1.44–4.02) | |

[a]Adjusted for age at interview, number of abortions (except for Lee et al), age at first child; [b]test for interaction; [c]odds ratios estimated on six data sets (Lê et al excluded); [d]odds ratios estimated on five data sets (Lê et al and Lee et al excluded).

with lifetime BCMA are higher for women with a family history of breast cancer compared with women without. Consequently, combined analyses lead to an increasing familial risk as lifetime BCMA increases except in the last category in which a slight decrease is observed. For duration of BCMA less than 28 years, between 28 and 32 years, between 33 and 37 years and greater than 37 years, $OR_{FR}$ = 1.89, 2.16, 2.72 and 2.21 with WM and 2.03, 2.12, 2.71 and 2.38 with GS respectively.

### DISCUSSION

The findings of this study with regard to the effects of reproductive life factors on the risk of breast cancer are in agreement with previous studies (Kelsey and Horm-Ross, 1993). An increased risk of breast cancer was found to be associated with an early age at menarche, a late age at first childbirth, nulliparity, premenopausal status and increasing durations of both pre-first-childbirth BCMA and lifetime BCMA. Results were consistent from study to study and risk factors seemed similar wherever the studies were conducted.

Exposure to menstrual activity has been used as a surrogate for assessing BCMA, which is mainly controlled by oestrogens. In assessing BCMA before first childbirth and over the entire reproductive life, we did not account for periods of oral contraceptive use. However, this may not lead to inaccurate measurement because an increase in BCMA has been found in the later weeks of the oral contraceptive cycle. Indeed, results of two studies suggested that total BCMA may be very similar over an oral contraceptive cycle and a normal cycle (Pike et al, 1993).

The accuracy of the measurement we have used to assess BCMA could, however, still be challenged and the overall cumulative number of menstrual cycles may be a better measurement. Indeed, experimental evidence indicates that BCMA is maximal in the luteal phase of the cycle (Anderson et al, 1982; Ferguson and Anderson, 1982). Moreover, the luteal phase appears to be less variable than the follicular phase, leading to women with short cycles spending relatively more time in the luteal phase and consequently in mitotic activity, than do women with longer cycles. However, menstrual cycle length was not available in most studies and even if it was, retrospective reports of menstrual bleeding have been shown to be unreliable (Whelan et al, 1994).

Lifetime BCMA showed a higher increase in risk than pre-first-childbirth BCMA. Moreover, this effect is consistent across studies with similar point estimates of odds ratios, and higher values for longer exposure making BCMA a relevant risk factor.

When interactions were investigated, a slight increasing familial risk was observed with an increasing number of children ($P = 0.17$), an increasing age at first childbirth ($P > 0.20$) and an increasing lifetime BCMA ($P > 0.2$). However, none of these interactions was significant. No modification in familial risk was found with age at menarche, and there was also no clear pattern with pre-first-childbirth BCMA nor menopause characteristics.

Some studies have investigated variations in familial risk according to reproductive factors, whereas other studies have investigated variations in reproductive risk factors according to familial factors. The tests performed to detect variations in risks were similar whichever of these two approaches was used. We have chosen to present and comment on results such as the effect of a family history stratified by reproductive factors as we were interested in the modifications of the familial risk due to reproductive factors. No interaction between familial factor and reproductive factors was significant, but it is possible that this is due to lack of power to detect interactions even with large sample sizes.

The two methods used to perform the combined analyses led to similar results in which data were complete. Moreover none of the tests of heterogeneity of effects between studies were not significant. From these two observations, we conclude that there are not important residual differences among the study results. The GS method allowed blank categories to contribute information to the estimation of the combined effects, whereas in the WM method such data cannot be used. This led to small differences in some of the estimates and confidence intervals.

The measurement of a family history of breast cancer was not homogeneous from study to study. Four studies recorded information in first- and second-degree relatives (Lê et al, 1984; Clavel et al, 1991; Luporsi, 1988; Lifanova, unpublished). Two studies recorded information in first- and second-degree relatives but not in grandmothers (Richardson et al, 1991; Lee et al, 1992) and one study recorded information in first-degree relatives only (Rohan et al, 1988). The heterogeneity in definition of family history could substantially explain the observed difference in familial risk estimates from study to study. This difference affects the precision of the combined familial risk estimate leading to a decrease in power for detecting a variation in this risk by reproductive factors. Thus, the risk estimated from the combined analyses measured the familial risk of breast cancer without a precise definition of the familial relationship. The heterogeneity in the method of measuring family history might have induced errors in the interaction estimations if genetic susceptibility differs according to type of familial relationship with an affected relative, and if the reproductive factors effect differs according to the type of genetic susceptibility. The occurrence of both conditions is necessary for there to be errors in the estimation of the interaction term. Byrne et al (1991) found that different factors could modify in different directions the effects of an affected mother and the effects of an affected sister. However, aside from the fact that type of affected relatives will probably not define a homogeneous group of genetic susceptibility, the number of cases in our study was not large enough to subdivide subjects with a family history of breast cancer according to the type of affected relatives. Further studies including a larger number of subjects could investigate the variation of the interaction according to the type of relationship of affected relative.

'No family history' in this study included 'unknown family history'. This could bias the results if cases were more aware of such a history than controls. In several studies, however, cases and controls had a similar proportion of relatives with an unknown cancer status and this proportion is small (among first-degree relatives: Lê et al 1.5%; Richardson et al 3%; Clavel et al 2%; Luporsi 3%). Also, although this bias might affect the estimation of the relative risk for the main effect of family history, there is no reason to assume that it would vary according to the reproductive factors.

Familial effects have been described as increasing in younger women. A difference in familial effects on breast cancer risk according to the age of subjects could introduce confounding in the interactions studied, even with adjustment for age, and a three-dimensional interaction (family history – reproductive factor – age of subjects) may be needed. However, a recent meta-analysis showed a breast cancer risk associated with a family history of breast cancer in first-degree relatives of 1.9 (1.8–2.8) in women

 © Cancer Research Campaign 1998

older than 50 years and of 2.4 (2.2–2.7) in women younger than 50 years (Pharoah et al, 1997). The difference in familial risks did not appear large. Nevertheless, as before, further studies including a large number of subjects could investigate the effect of subjects' age on the possible interactions between reproductive life factors and family history.

Among eight studies that have investigated the variation in breast cancer risk associated with age at menarche by family history, three found such a variation, in contrast with our study in which no difference has been found between groups (Bain et al, 1980; Brinton et al, 1982; Negri et al, 1988; Malone and Daling, 1992; Parazzini et al, 1992; Andrieu et al, 1993; Sellers et al, 1993; Colditz et al, 1996). In previous studies, the variation always reflected the same trend, namely an increased risk associated with a late age at menarche for women with a family history of breast cancer and a decreased risk associated with a late age at menarche for women without family history (Bain et al, 1980; Parazzini et al, 1992; Malone and Daling, 1992). None of the other studies found an increased risk associated with a late age at menarche among women with a family history and they failed to observe a decreasing risk with late age at menarche such as was observed among women without a family history (Brinton et al, 1982; Negri et al, 1988; Andrieu et al, 1993; Sellers et al, 1993; Colditz et al, 1996).

Seven epidemiological studies have researched an interaction between parity and family history of breast cancer (Bain et al, 1980; Negri et al, 1988; Parazzini et al, 1992; Sellers et al, 1992; 1993; Colditz et al, 1993; 1996). Four studies out of seven did not observe a variation in familial risk according to number of children (Bain et al, 1980; Sellers et al, 1992; 1993; Colditz et al, 1993). In the three others, no protection from multiple births was observed when compared with nulliparity among women with a family history of breast cancer (Negri et al, 1988; Parazzini et al, 1992; Colditz et al, 1996). In the present study, no clear pattern emerges in the variation of familial risk between nulliparous and parous women. However, familial risk seems to increase slightly ($P = 0.17$) for women with a high parity (three and more children) compared with women with a low parity (one or two children).

Age at first full-term pregnancy has been studied in eight published studies. No evident increase in risks according to age at first childbirth has been found among women with a family history of breast cancer in three studies (Negri et al, 1988; Byrne et al, 1991; Colditz et al, 1993). In two others, a similar increase in risk with age at first childbirth has been observed (Brinton et al, 1982; Parazzini et al, 1992). In the three remaining studies, in agreement with the tendency of our finding, an increase in risk has been described that is stronger among women with a family history than among women without a family history (Dupont and Page, 1987; Sellers et al, 1992; Colditz et al, 1996).

Parity and first full-term pregnancy have been suggested to have antagonistic main effects on breast cells. Indeed, animal models suggest that their effects are a combination of increased mitotic activity during the first two trimesters of pregnancy with the counteracting effect of breast cells differentiation during the last trimester (Russo and Russo, 1980). If different types of susceptibility are varyingly sensitive to the antagonistic effects of full-term pregnancy and parity on breast cells, then one could partially explain the difficulty in detecting a clear pattern in familial risk variations and consequently the observed differences in published studies.

To our knowledge, only two studies have examined interaction between familial risk and BCMA (Bain et al, 1980; Colditz et al, 1993). These two analyses were performed in the Nurses' Health Study. The authors used the absolute interval between menarche and menopause as BCMA. In agreement with the trend of our results, they showed an increased familial risk among women who menstruated for more than 35 years in the first report, based on prevalent breast cancer cases (Bain et al, 1980), but not in the prospective data (Colditz et al, 1993). Obviously, longer mitotic activity increases the probability of damaging DNA and of converting DNA damage into mutation, and may have a critical effect on cells predisposed to become malignant because of inherited mutations. In the present study, a difference in the increase in risks associated with BCMA between women with and without a family history of breast cancer is not observed for pre-first-childbirth BCMA but slight difference was found for lifetime BCMA. This difference appears for BCMA durations over 28 years. An increased duration of BCMA might be associated with a slight increased risk of breast cancer for genetically susceptible women. Further studies including a very large number of cases may verify the existence of this plausible effect.

A possible weak influence of reproductive and menstrual factors on the familial risk emerges from the present study. Further studies including larger number of subjects could increase power in detecting interactions and permit the investigation of the variation of the familial-reproductive factors interactions according to the type of relationship of affected relative and the age of subjects.

### ACKNOWLEDGEMENT

This study was made possible by an MRC-INSERM exchange grant.

### REFERENCES

Adami HO, Hansen J, Jung B and Rimsten A (1980) Familiality in breast cancer: a case–control study in a Swedish population. *Br J Cancer* **42**: 71–77

Anderson TJ, Ferguson DJP and Raab GM (1982) Cell turnover in the «resting» human breast: influence of parity, contraceptive pill, age and laterality. *Br J Cancer* **46**: 676–682

Andrieu N, Demenais F and Martinez M (1988) Genetic analysis of human breast cancer: implications for family study designs. *Gen Epidemiol* **5**: 225–233

Andrieu N, Clavel F, Auquier A, Lê MG, Gairard B, Piana L, Brémond A, Lansac J, Flamant R and Renaud R (1993) Variations in the risk of breast cancer associated with a family history of breast cancer according to age at onset and reproductive factors. *J Clin Epidemiol* **46**: 973–980

Andrieu N, Duffy S, Rohan T, Lê MG, Luporsi E, Gerber M, Renaud R, Zaridze DG, Lifanova Y and Day N (1995) Familial risk, abortion and interactive effect on the risk of breast cancer – a combined analysis of six case–control studies. *Br J Cancer* **72**: 744–751

Bain C, Speizer FE, Rosner B, Belanger C and Hennehens CH (1980) Family history of breast cancer as a risk indicator for the disease. *Am J Epidemiol* **111**: 301–308

Brinton L, Hoover R and Fraumeni J (1982) Interaction of familial and hormonal risk factors for breast cancer. *J Natl Cancer Inst* **69**: 817–822

Byrne C, Brinton LA, Haile RW and Schairer C (1991) Heterogeneity of the effect of family history on breast cancer risk. *Epidemiology* **2**: 276–284

Claus EB, Risch NJ and Thompson WD (1991) Genetic analysis of breast cancer in the Cancer and Steroid Hormone Study. *Am J Hum Gen* **48**: 232–242

Clavel F, Andrieu N, Gairard B, Bremond A, Piana L, Lansac J, Breart G, Rumeau-Rouquette C, Flamant R and Renaud R (1991) Oral contraception and breast cancer: a French case–control study. *Int J Epidemiol* **20**: 32–38

Colditz GA, Willett WC, Hunter DJ, Stampfer MJ, Manson JE, Hennekens CH, Rosner BA and Speizer FE (1993) Family history, age, and risk of breast cancer. *J Am Med Assoc* **270**: 338–343

Colditz GA, Rosner BA and Speizer FE (1996) Risk factors for breast cancer according to family history of breast cancer. *J Natl Cancer Inst* **88**: 365–371

© Cancer Research Campaign 1998

Demenais F, Martinez M, Bonaiti-Pellié C, Clerget-Darpoux F and Feingold N (1986) Segregation analysis of the Jacobsen data. *Gen Epidemiol* (Suppl.) 1: 49–54

Dupont WD and Page DL (1987) Breast cancer risk associated with proliferative disease, age at first birth, and a family history of breast cancer. *Am J Epidemiol* **125**: 769–779

Ewertz M, Duffy SW, Adami H-O, Kvale G, Lund E, Meirik O, Mellemgaard A, Soini I and Tulinius H (1990) Age at birth, parity and risk of breast cancer: a meta-analysis of 8 studies from the Nordic countries. *Int J Cancer* **46**, 597–603

Ferguson DJP and Anderson TJ (1982) Morphologic evaluation of cell turnover in relation to the menstrual cycle in the 'resting' human breast: influence of parity, contraceptive pill, age and laterality. *Br J Cancer* **46**: 177–181

Gelfand AE and Smith AFM (1990) Sampling-based approaches to calculating marginal densities. *J Am Stat Assoc* **85**: 398–409

Gilligan SB and Borecki IB (1986) Examination of heterogeneity in 200 Danish breast cancer pedigrees. *Gen Epidemiol* (suppl.) **1**: 67–72

Goldstein AM, Haile RWC, Hodge SE, Paganini-Hill A and Spence MA (1988) Possible heterogeneity in the segregation pattern of breast cancer in families with bilateral breast cancer. *Gen Epidemiol* **5**: 121–133

Goldstein AM and Amos CI (1990) Segregation analysis of breast cancer from the Cancer and Steroid Hormone Study: Histologic Subtypes. *J Natl Cancer Inst* **82**: 1911–1917

Hall JM, Lee MK, Morrow J, Anderson L and King MC (1990) Linkage of early onset of familial breast cancer to chromosomes 17 q 21. *Science* **250**: 1684–1689

Iselius L, Slack J, Littler M and Morton NE (1991) Genetic epidemiology of breast cancer in Britain. *Ann Hum Gen* **55**: 151–159

Kelsey JL and Horm-Ross PL (1993) Breast cancer. Magnitude of the problem and descriptive epidemiology. *Epidemiol Rev* **15**: 7–16.

Lê MG, Bachemot A, Doyon F, Kramar A and Hill C (1984) Oral contraceptive use and breast cancer or cervical cancer: preliminary results of a French case–control study. In *Hormones and Sexual Factors in Human Cancer Aetiology* Wolff J-P and Scott JD (eds), pp. 139–147. Elsevier Science: Amsterdam

Lee HP, Gourley L, Duffy SW, Estève J, Lee J and Day NE (1992) Risk factors for breast cancer by age and menopausal status: a case–control study in Singapore. *Cancer Cause Cont* **3**: 313–322

Luporsi E (1988) Breast cancer and alcohol. PhD thesis, University of Paris-Sud

Malone KE and Daling JR (1992) Family history as a modifier of breast cancer risk factors. *Am J Epidemiol* **136**: 964

Narod SA, Feunteun J, Lynch HT, Watson P, Conway T, Lynch J and Lenoir G (1991) Familial breast-ovarian cancer chromosome 17q 12–23. *Lancet* **338**: 82–83

Negri E, La Vecchia C, Bruzzi P, Dardanoni G, Decarli A, Palli D, Parazzini F and Del Turco MR (1988) Risk factors for breast cancer: pooled results from three Italian case-control studies. *Am J Epidemiol* **128**: 1207–1215

Newman B, Austin MA, Lee M and King MC (1988) Inheritance of human breast cancer: evidence for autosomal dominant transmission in high-risk families. *Proc Natl Acad Sci USA* **85**: 3044–3048

Olsson H, Olsson ML, Kristoffersson U and Ranstam J (1985) Risk factors of breast cancer in relation to a family history of breast cancer in southern Sweden. In *Familial Cancer,* Muller HJ and Weber W (eds), pp. 34–35. Springer: Basle

Parazzini F, La Vecchia C, Negri E, Franceschi S and Bocciolone L (1992) Menstrual and reproductive factors and breast cancer in women with family history of the disease. *Int J Cancer* **51**: 677–681

Pharoah P, Day N, Easton D, Duffy S and Ponder B (1997) Family history and the risk of breast cancer: a systematic review and meta-analysis. *Int J Cancer* **71**: 800–809

Pike MC, Spicer DV, Dahmoush L and Press MF (1993) Estrogens, progestogens, normal breast cell proliferation and breast cancer risk. *Epidemiol Rev* **15**: 17–35

Richardson S, Gerber M and Pujol H (1985) Familial risk in a case–control study on breast carcinoma. In *Familial Cancer*, pp. 31–33, Muller HJ and Weber W (eds), Springer: Basle,

Richardson S, Gerber M and Cenee S (1991) The role of fat, animal protein and vitamin consumption in breast cancer. A case–control study in Southern France. *Int J Cancer* **48**: 1–19

Rohan T, McMichael AJ and Baghurst PA (1988) A population-based case–control study of diet and breast cancer in Australia. *Am J Epidemiol* **128**: 478–489

Russo J and Russo IH (1980) Susceptibility of the mammary gland to carcinogenesis. *Am J Pathol* **100**: 497–512

Sattin RW, Rubin GL, Webster LA, Huezo CM, Wingo PA, Ory HW and Layde PM (1985) The cancer and steroid hormone study. Family history and the risk of breast cancer. *J Am Med Assoc* **253**: 1908–1913

Sellers AS, Kushi LH, Potter JD, Kaye SA, Nelson CL, McGovern PG and Folsom A (1992) Effect of family history, body-fat distribution, and reproductive factors on the risk of postmenopausal breast cancer. *N Engl J Med* **326**: 1323–1329

Sellers TA, Potter JD, Severson RK, Bostick RM, Nelson CL, Kushi LH and Folsom AR (1993) Difficulty becoming pregnant and family history as interactive risk factors for postmenopausal breast cancer: the Iowa Women's Health Study. *Cancer Caus Contr* **4**: 21–28

Williams WR and Anderson DE (1984) Genetic epidemiology of breast cancer: segregation analysis of 200 Danish pedigrees. *Gen Epidemiol* **1**: 7–20

Whelan EA, Sandler DP, Root JL, Smith KR and Weinberg CR (1994) Menstrual cycle patterns and risk of breast cancer. *Am J Epidemiol* **140**: 1081–1090

Woolf B (1955) On estimating the relation between blood group and disease. *Ann Hum Gen* **12**: 251–253

Wooster R, Neuhausen SL, Mangion J, Quirk Y, Ford D, Collins N, Nguyen K, Seal S, Tran T, Averill D et al (1994) Localization of a breast cancer susceptibility gene, BRCA2, to chromosome 13q12–13. *Science* **265**: 2088–2090

© Cancer Research Campaign 1998